# Innovations in invasive parasite control: enhancing nest treatment techniques to combat the threat of the avian vampire fly *Philornis* *downsi* in Galapagos

**DOI:** 10.3389/fcosc.2025.1591266

**Published:** 2025-11-25

**Authors:** Barbara Kofler, Merlin Mauchamp-Fessl, Cristian Poveda-Pazmiño, Charlotte E. Causton, Sabine Tebbich, Birgit Fessl

**Affiliations:** 1 Department of Behavioral Biology and Cognition, University of Vienna, Vienna, Austria; 2 Charles Darwin Research Station, Charles Darwin Foundation, Puerto Ayora, Santa Cruz, Galapagos, Ecuador

**Keywords:** self-fumigation, spraying, Darwin*’*s finches, parasite control, conservation, invasive species, *Philornis downsi*

## Abstract

The invasive parasitic nest fly *Philornis downsi* poses a severe threat to the conservation of Galapagos’ endemic landbirds, including Darwin’s finches. Therefore, the development of effective stop-gap methods is required to mitigate its harmful impact until long-term solutions are found. This study aims to enhance the usability of two insecticide-based control methods designed to reduce fly infestation: 1) Self-fumigation during which birds incorporate insecticide-treated nesting material into their nests, and 2) the Spritz technique, which involves spraying insecticide around the nest entrance to prevent female flies from entering nests to lay eggs. To improve the efficacy and broaden the applicability of self-fumigation across species, we tested the effects of two insecticides using this method (Cyromazine and Permacap CS®) on per-nest *P. downsi* abundance and fledging success in three Darwin’s finch species, Small Ground-finch (*Geospiza fuliginosa*), Small Tree-finch (*Camarhynchus parvulus*), and Green Warbler-finch (*Certhidea olivacea*). We employed a stepwise approach to optimize method efficacy through variation in the insecticide used and its dosage, dispenser setup, and the type of material offered to birds. Cyromazine was effective in reducing *P. downsi* abundance, but did not result in increased fledging success. Permacap-treated materials at 0.5% and 1% concentrations significantly increased fledging success. Four nesting materials offered in dispensers placed 4 m high were widely accepted by Darwin’s finches. For the Spritz technique, we also tested the effects of the two Permacap concentrations on *P. downsi* abundance and fledging success over two consecutive breeding seasons. Using a novel, lightweight, and pole-compatible spraying device with 0.5% Permacap, fledging success improved significantly across all tested finch species, while minimizing nest abandonment risk. These methods offer immediate, effective solutions for *P. downsi* control, and for improving fledging success in Darwin’s finches, potentially reducing extinction risks for some of the Galapagos’ most threatened species, and marking a critical step in preserving the archipelago’s unique avian diversity.

## 1 Introduction

Invasive alien parasites and pathogens pose a serious threat to the survival of naïve host species that lack effective defense mechanisms (Allison, 1982; Knutie et al., 2014; Lymbery et al., 2014; Dunn and Hatcher, 2015; Vilcinskas, 2019). Endemic species in island ecosystems, having evolved in isolation, are especially susceptible to biological invasions as they often have small populations that are vulnerable to rapid extinction (Wikelski et al., 2004; Bellard et al., 2016; Russell et al., 2017). The Galapagos archipelago is known as one of the world’s most pristine refuges for endemic avian fauna (Swarth, 1934; González et al., 2008; Cooke et al., 2019). In recent decades, introduced species have contributed to the decline of several bird populations (O’Connor et al., 2010b; Dvorak et al., 2012; Cimadom et al., 2014; Fessl et al., 2019; Jiménez-Uzcátegui et al., 2019). Among these, the parasitic nest fly *Philornis downsi* (Diptera: Muscidae), known as the avian vampire fly, is considered particularly problematic. Although, records were found from the 1960’s (Fessl et al., 2018), the parasite’s impact on the endemic and native avifauna was only recognized in the late 1990’s, when larvae were first found in bird nests (Fessl et al., 2001; Fessl and Tebbich, 2002). In Galapagos, *P. downsi* parasitizes nearly all nesting landbird species, including 12 of 17 endemic Darwin’s finch species (Fessl et al., 2018, S. A. Knutie, pers. comm.). The non-parasitic adult flies lay their eggs in bird nests, where the semi-hematophagous larvae feed on the blood and tissue of nestlings after hatching (O’Connor et al., 2010a; Lincango et al., 2015; Common et al., 2020). *Philornis downsi* parasitism can cause anemia, reduction in hemoglobin concentration, wounds, and deformation of nasal openings, leading to impaired nestling growth and survival (Dudaniec et al., 2006; Fessl et al., 2006; Kleindorfer and Sulloway, 2016; Knutie et al., 2016). Research has consistently shown that *P. downsi* parasitism results in high nestling mortality (16-100%) across most studied host species (Kleindorfer and Dudaniec, 2016) and has contributed to declines in several Darwin’s finch populations, including Small Tree-finch (*Camarhynchus parvulus*) and Green Warbler-finch (*Certhidea olivacea*), two focal species in this study (Fessl et al., 2010, 2019; Dvorak et al., 2012; Lawson et al., 2017; Cimadom et al., 2019). As a result, *P. downsi* is regarded as a major threat to the Galapagos avifauna and exemplifies the harmful effects an invasive, generalist parasite can have on bird populations in a fragile ecosystem.

Finding effective methods to control this parasite is crucial to safeguard the unique avian fauna of the archipelago (Causton et al., 2013; Fessl et al., 2018). Biological control using natural enemies from the fly’s native range is considered a promising option for controlling *P. downsi* populations in the long-term; however, this method is still under development (Bulgarella et al., 2017; Boulton et al., 2019; Ramirez et al., 2022). In the meantime, stop-gap measures are crucial for protecting species, sub-species, or island populations at risk of extinction. The most immediate solution is the treatment of nests with low toxicity insecticides and three methods are under trial: injection, spray (Spritz), and self-fumigation.

### 1.1 Application methods

The injection method involves using a syringe at the end of a pole to apply the insecticide directly into the nest base, where fly larvae reside during the day (Cimadom et al., 2019; Tebbich et al., 2019). This method is logistically challenging, limited to accessible nests, and requires skilled personnel to apply the product safely while minimizing risks to eggs, nestlings, as well as to conservation workers (Causton et al., 2019; Cimadom et al., 2019; Anchundia et al., 2024).

Self-fumigation offers a viable alternative, particularly for nests that are out of reach and for threatened bird species with patchy distributions over large areas (Knutie et al., 2014; Bulgarella et al., 2020). This approach involves providing insecticide-impregnated nesting material to birds in dispensers during the nesting season, which the birds incorporate into their nests. Self-fumigation with cotton significantly reduced *P. downsi* larval abundance in three Darwin’s finch species (Knutie et al., 2014), and insecticide-treated feathers enhanced hatchling survival in the Forty-spotted Pardalote (*Pardalotus quadragintus*) threatened by *Passeromyia longicornis* (Alves et al., 2021). Follow-up trials in Galapagos, building on the work of Knutie et al. (2014), targeted Darwin’s tree finches, including the critically endangered Mangrove Finch (*Camarhynchus heliobates*) and the Medium Tree-finch (*Camarhynchus pauper*). These trials yielded inconclusive results: either insufficient cotton was collected to ensure efficacy, or the finches did not visit the dispensers at all (Causton et al., 2020).

A third technique, which we call the Spritz technique, acts as a preventive method and involves spraying insecticide around the outside of the nest entrance to repel or eliminate flies as they attempt to enter. This method was first developed to protect Ridgway’s Hawk (*Buteo ridgwayi*) from the subcutaneous parasite *Philornis pici* in the Dominican Republic (M. Quiroga, pers. comm.), and was considered promising for use in Galapagos, based on observations that *P. downsi* flies often walk around the nest entrance before entering to lay eggs (Pike et al., 2021). Furthermore, laboratory trials demonstrated that brief exposure to nesting material sprayed with 1% Permacap CS® (BASF, USA) effectively killed *P. downsi* flies within 1–3 minutes (CDF, unpubl. data). This technique is likely most effective during the incubation phase, as *P. downsi* targets hatchlings and oviposits in some nests as early as mid-incubation (Cimadom and Tebbich, 2021; Mosquera et al., 2022).

### 1.2 Insecticides

Historically, the synthetic pyrethroid permethrin has been used with the techniques aforementioned to protect nestlings from *P. downsi* parasitism in Galapagos in the form of an emulsifiable concentrate (EC) (Permectrin II® Bayer, USA) or a microencapsulated concentrate (Permacap CS®). The targeted injection of 1% permethrin EC into nest bases has proven to be highly effective in controlling fly larvae in nests in Galapagos (Fessl et al., 2006; Koop et al., 2013; Knutie et al., 2014, 2016, 2024; Kleindorfer and Sulloway, 2016). Nevertheless, spraying of the entire nest with 1% permethrin EC may result in negative effects on the long-term breeding success of passerines, as suggested by a study on Zebra Finch (*Taeniopygia guttata*) nestlings (Bulgarella et al., 2020). Similarly, López-Arrabé et al. (2014) observed reduced growth in Pied Flycatcher (*Ficedula hypoleuca*) nestlings, along with elevated oxidative stress in both nestlings and brooding females after nests and nest boxes were sprayed with a pyrethroid-based insecticide, that included tetramethrin, permethrin and a synergist (piperonyl butoxide). As a safeguard, more recent trials in Galapagos (Mosquera et al., 2022; Pike et al., 2023; Anchundia et al., 2024) have used the controlled-release formulation of permethrin, Permacap, which gradually releases the active ingredient, reducing dermal exposure and lowering peak concentrations (Causton and Lincango, 2014; Bueno et al., 2021) and thereby minimizing potential negative effects on nestling development.

Another option under investigation is the use of Cyromazine, an insect growth inhibitor, that has been shown to significantly reduce the number of fly larvae per nest (Causton et al., 2019, 2020). More selective than permethrin, it targets dipteran insects by inhibiting larval growth and development (Van De Wouw et al., 2006). Cyromazine is considered to have low avian toxicity (Bueno et al., 2021), and preliminary applications in Galapagos, which involved spraying 0.4 g/L Cyromazine solution onto the inner nest layer after temporarily removing eggs or nestlings, indicated no adverse short-term effects for birds (Causton et al., 2020).

The effectiveness and safety of using Cyromazine or Permacap to reduce parasite intensity in bird nests depend on both the application method and concentration of the insecticide used, with important knowledge gaps and practical challenges remaining – particularly regarding the effectiveness of self-fumigation with Cyromazine and Permacap, the difficulty of accessing nests to use the Spritz technique, and the difference in response to the Self-fumigation method and Spritz technique across bird species (Causton et al., 2019; Bueno et al., 2021).

### 1.3 Study aims

This study aimed to address methodological uncertainties and identify practical short-term management tools to reduce *P. downsi* abundance in Darwin’s finch nests. To this end, we tested and evaluated the effectiveness of two methods: (1) an improved Self-fumigation technique, previously tested with unprocessed cotton (Knutie et al., 2014; Causton et al., 2020), and (2) a newly developed Spritz technique, designed as a non-invasive alternative to direct nest injection (Cimadom et al., 2019; Tebbich et al., 2019).

For the Self-fumigation technique, we assessed the attractiveness of different nesting materials to finches, and tested the effectiveness of two insecticidal compounds, Cyromazine and Permacap, in reducing *P. downsi* abundance and increasing nesting success. We also examined how dispenser number and placement influenced the method’s efficiency as a self-administered treatment tool.

The Spritz technique was developed as a complementary approach for endangered or small populations where targeted nest treatment is required to minimize parasite load. Using two concentrations of Permacap (0.5% and 1%), we tested the method’s effectiveness in reducing *P. downsi* abundance and improving fledging success, while also monitoring nest abandonment rates to ensure the method’s suitability for conservation application.

## 2 Materials and methods

### 2.1 Study site

The study was conducted in the humid highlands of Santa Cruz Island, Galapagos, at the “Los Gemelos” site within the Galapagos National Park (S 00°37′20′′–45′′ W 90°23′00′′–15′′, 500–600 m a.s.l.). The research area was situated within an approximately 11 ha patch of restored cloud forest with ongoing management to control invasive plant species (Jäger et al., 2024). The forest is dominated by the endemic tree species *Scalesia pedunculata* (Asteraceae) and is therefore referred to as the Scalesia zone. The area has been invaded by several introduced plant species, including *Rubus niveus* (blackberry, Rosaceae), *Tradescantia fluminensis* (river spiderwort, Commelinaceae), *Cestrum auriculatum* (sauco, Solanaceae), and *Piper peltatum* (Piperaceae). This forest supports a diverse Galapagos landbird community, including Small Tree-finch, which has experienced local population declines, and Green Warbler-finch, listed as vulnerable (VU) in the IUCN Red List (Dvorak et al., 2012; Cimadom et al., 2014, 2019; Fessl et al., 2019; Heyer et al., 2021; IUCN, 2024). The self-fumigation experiment was conducted in 2022 and 2023 within a ~ 4.5 ha plot located at the lower boundary of the study area. The Spritz experiment was carried out in 2023 and 2024 in a separate plot several hundred meters away, ensuring experimental independence.

### 2.2 Nest search and monitoring

Nest search and monitoring were conducted daily between 06:00 and 12:00 throughout the bird breeding seasons from January to April, following the protocol outlined by Cimadom et al. (2014). In 2024, nest searches ended in March, with monitoring continuing into April. All occurring Darwin’s finch species (Small Ground-finch *Geospiza fuliginosa*, Medium Ground-finch *G. fortis*, Small Tree-finch, Large Tree-finch *Camarhynchus psittacula*, Woodpecker Finch *C. pallidus* and Green Warbler-finch) were monitored at intervals tailored to the breeding stage to minimize disturbance and ensure accurate data collection: every 5 days during nest building, every 3 days during incubation, and every 2 days during feeding. From incubation status onwards, an endoscopic camera (dnt Findoo; Depstech-View) mounted on a 1.5 m pole, extendable to 12 m, was used to inspect nests and document breeding onset, clutch size, hatching dates, nestling number, and fledging success. Nestling age was determined using monitoring dates in combination with nestling appearance. For nests found during the late feeding stage where nestlings successfully fledged, nestling age was assumed to be 13 days, based on the mean fledging age calculated across species in 2023. After confirming the cessation of nest activity, each monitored nest was collected in a sealed plastic bag, taken to the laboratory at the Charles Darwin Research Station (CDRS), and dismantled on the same day. *Philornis downsi* abundance per nest was quantified as the total number of specimens (larvae, pupae, and empty puparia) within a nest.

### 2.3 Self-fumigation

#### 2.3.1 Effectiveness of Cyromazine

**Figure 1:**
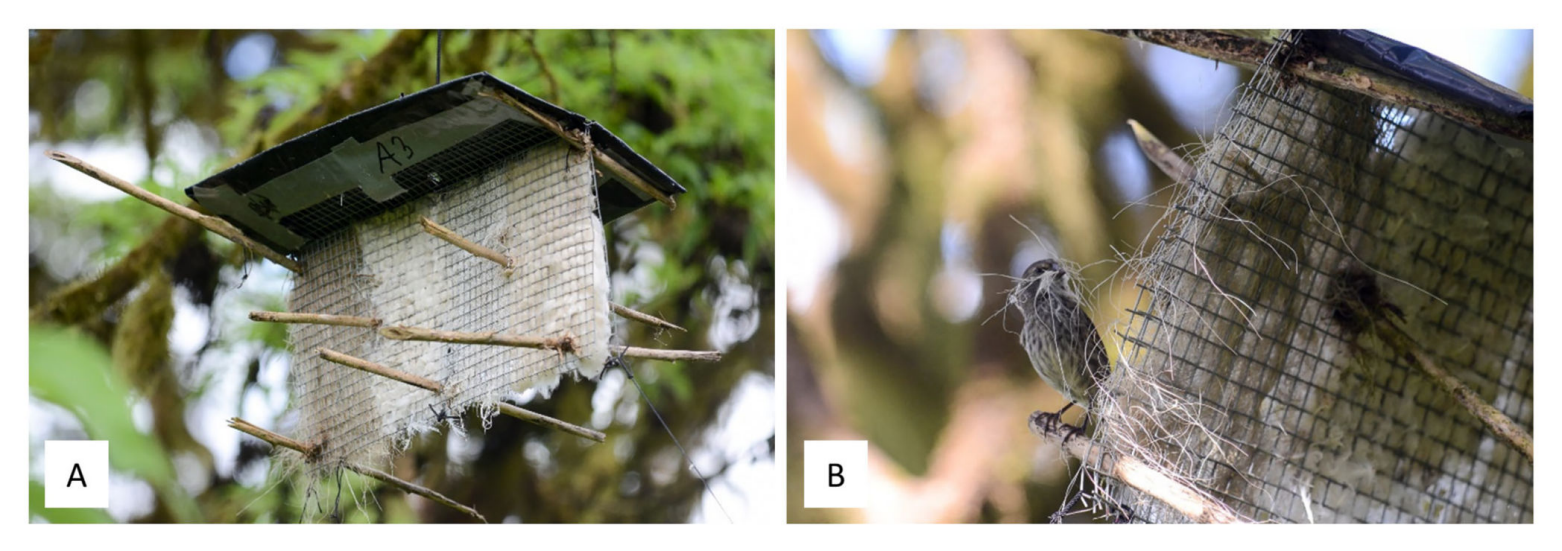
**(A)** Nest material dispenser providing four different materials (left to right: sisal, feathers, cotton fibers, kapok) at a height of 4 m. Sticks were threaded through the mesh to create perches for the birds, facilitating material collection and the materials were protected from rain by a plastic roof. **(B)** A Small Ground-finch taking sisal from the dispenser. Photos: BK.

To offer birds insecticide-treated nesting materials, eighteen pairs of flat dispensers (25 × 60 cm, 1 cm mesh) were installed in the study plot. The dispenser design followed Knutie et al. (2014), with sticks threaded through the mesh to create perches and a plastic roof to protect the materials from rain. Dispensers were placed 50 m apart, with one at 4 m and the other at 1.5 m in height. The 50 m spacing was selected to balance coverage and field effort, and was informed by Supplementary Data from Knutie et al. (2014) showing that only nests within 25 m of dispensers contained material. Each dispenser was filled with six material types: hemp fibers, kapok, cotton fibers, feathers, sisal fibers (all commercially available), and coconut fibers, sourced locally and sun-dried (see Figure 1 for an example of the design). Materials were not sterilized, as they were freshly purchased or processed in clean conditions and stored in sealed bags. Materials were weighed and equally distributed by volume in the dispensers (Supplementary Material Table S1).

A concentration of 0.75 g Cyromazine/L was prepared using 1 g of Trigard® powder (Syngenta, 75% Cyromazine) diluted in 1 L of drinking water. This concentration was higher than the Cyromazine dilution recommended for nest injection (0.4 g/L, Causton et al., 2019) to account for the potentially lower insecticide concentrations in self-fumigated nests. The Cyromazine dilution was applied evenly to the dispenser material using a commercially available spray bottle at a rate of 1 mL/1 g of material and applied at ~10–15 cm distance. Dispensers were refilled and retreated every 3 weeks, based on laboratory tests evaluating the efficacy of Cyromazine on the development of *P. downsi* larvae (CDF, unpubl. data). If any material type was depleted, dispensers were replenished with fresh, dry materials and treated between scheduled intervals to ensure constant availability of all materials. The material used for refilling the dispensers was weighed directly in the field using a spring balance with a precision of 0.1 g.

#### 2.3.2 Effectiveness of Permacap

Building on the experience of the first season, several adjustments were implemented in the trial of the following season. Dispensers were placed in the same locations as 2022. Based on material usage being greater in dispensers positioned higher in the canopy (Supplementary Material Table S2), the number of dispensers was halved in 2023, with a single dispenser per point placed at a height of 4 m. Of the six materials used in 2022, only the four that were most preferred by birds (sisal fibers, feathers, cotton fibers, and kapok) were included in the 2023 study (Supplementary Material Table S1). For this experiment, Permacap was used to treat material. Initially, a 0.5% Permacap dilution in drinking water was used following protocols from previous studies involving nest injections and laboratory experiments (Causton and Lincango, 2014; Causton et al., 2019; Cimadom et al., 2019; Mosquera et al., 2022; Pike et al., 2023; Anchundia et al., 2024). However, because of the difference in technique, and to ensure efficacy, the concentration was increased to 1% two weeks later. The nests constructed with material that had been treated with 0.5% Permacap were analyzed separately.

Pre-prepared bags of 1, 2, 5, and 10 g of material were used to replace missing material during the refilling stage. The refilling interval was shortened to 2 weeks based on findings that demonstrated that Permacap at 0.5% achieved 96.9% larval mortality after 7 days and 67.7% after 2 weeks under semi-natural conditions (Causton et al., 2019).

#### 2.3.3 Quantification of dispenser materials from nests

Following the extraction of *P. downsi*, each nest was thoroughly inspected to identify and retrieve all dispenser materials. Dispenser material collected from nests was sorted by material type and nest identity, stored in small cardboard cups and dried for 24 h at 60 °C in a drying chamber. The material was weighed to the nearest 0.001 g. Since the insecticide was applied uniformly to the dispenser surface, the amount of insecticide on the material depended on its volume. Densities of the natural fibers (Supplementary Material Table S3), were used to convert weight to volume (Bisanda and Ansell, 1991; Zheng and Wang, 2014; Tesfaye et al., 2017; Elmogahzy and Farag, 2018; Ramesh, 2018; Mishra and Gautam, 2020).

### 2.4 Spritz technique

A custom-built spraying tool was developed to apply insecticide to the outer surface of the nest material around the dome-shaped nest entrance. It featured an adjustable valve and was connected to a Makita battery-powered compressor (Supplementary Figure S1).

Preliminary trials with water determined that the application of 5–6 mL of liquid was sufficient to cover the nest entrance without soaking the material. A small video camera (Ubox), connected to a smartphone, was mounted under the valve for directional control. The device was attached on a 1.5 m carbon pole, with extensions allowing treatment of nests up to 10 m high. A two-person team operated the device: one controlled the operation via the smartphone and activated the compressor, while the other positioned the spray head.

As a preventive measure against infestation, treatment was applied only during the incubation phase. Because Darwin’s finches are sensitive to disturbance during early incubation, treatment was delayed by three days after incubation was confirmed. Prior to spraying, nest activity was verified through observations lasting up to 40 min. Treatment was carried out when the incubating parent left and both parents were out of sight. Nests were filmed and their status confirmed before spraying. The filming and spraying process was quick, taking approximately 5 min. Nests were observed within 1 hour after treatment and again the following day to assess possible abandonment. If a nest appeared inactive, additional observations were made 24 and 48 hours later to confirm abandonment. Active nests continued to be monitored as previously described. Once activity ceased, nests were collected separately in sealed plastic bags and taken to the CDRS to assess *P. downsi* abundance.

In 2023, the treatment was applied to nests of three Darwin’s finch species: Small Ground-finch, Green Warbler-finch, and Small Tree-finch. A total of 25 nests were treated with 1% Permacap, and 24 nests received water as control. Additionally, four nests were treated with 0.5% Permacap. In 2024, the study focused on the Green Warbler-finch and Small Tree-finch, given their population declines and conservation relevance. That year, 23 nests were treated with 0.5% Permacap and 20 with water.

### 2.5 Statistical analyses

All statistical analyses were conducted using R version 4.3.1 (R Core Team, 2023). Data preprocessing was carried out using the “fe.re.tab” function (developed by R. Mundry), and continuous predictors were z-transformed to a mean of 0 and a standard deviation of 1 to facilitate model convergence (Schielzeth, 2010), prior to integration into generalized linear mixed models using the “glmmTMB” function from the eponymous package (version 1.1.8) (Brooks et al., 2017). Model diagnostic assessments were conducted using the “DHARMa” package (version 0.4.6) (Hartig, 2022). Collinearity among predictors was evaluated by calculating variance inflation factors (VIFs) with the “vif” function from the “car” package (version 3.1.2) (Fox and Weisberg, 2019), ensuring that VIFs remained below a threshold of 3. Each model was then compared to a null model, excluding the test predictor(s), using chi-squared ANOVA to evaluate model fit and statistical significance in alignment with the hypothesis being tested. For all analysis nests within 100 m of a dispenser point at the study plot’s edge were considered. To account for seasonal effects where appropriate, nests were assigned a day count based on the difference between season onset and incubation start. In Darwin’s finches, the incubation period typically lasts around 14 days, calculated from the first incubation observation to hatching (Cimadom et al., 2014). Therefore, for nests where eggs successfully hatched, the incubation onset was estimated by subtracting the nestling’s age and the standard incubation duration of 14 days. For nests with unknown incubation onset, the date of the first observed incubation activity was used.

An overview of all generalized linear models (GLMs) used in this study is provided in Supplementary Material (Supplementary Table S9).

#### 2.5.1 Dispenser material use

To assess variation in the prevalence of nests containing material and the quantity of dispenser material incorporated across study years and species, two models were used. Differences in the prevalence of nests containing material (response) across years and species were tested using a binomial GLM (N = 217) with a logit link function, including the year × species interaction as the test predictor and controlling for seasonal effects. Only “complete” nests (those that reached the incubation phase and were undamaged at collection) were included, focusing on the three most abundant species in the study area (Small Ground-finch, Small Tree-finch, and Green Warbler-finch) to ensure sufficient observations (>6) per year-species group. The full model explained significantly more variation in *P. downsi* abundance per nestling than the null model that excluded the test predictor (χ^2^ = 18.599, df = 5, p = 0.002). In a separate model, variation in dispenser material volume per nest (response) was analyzed as a function of the year × species interaction (test predictor) using a Gaussian model (N = 145), including only nests containing dispenser material. The seasonal effect was included as control predictor. The full model accounted for variation in the response significantly better than the null model that excluded the test predictor (χ^2^ = 86.227, df = 5, p < 0.001). *Post-hoc* comparisons were conducted with the “emmeans” function from the same named package (version 1.8.8) (Lenth, 2023).

#### 2.5.2 Effect of self-fumigation on *P. downsi* abundance

To analyze the effect of self-fumigation on *P. downsi* abundance, only nests from Small Ground-finch, Small Tree-finch, and Green Warbler-finch with hatched nestlings were included, as larvae mostly hatch during the feeding phase, though some nests may be infested prior to hatching (Cimadom et al., 2016; Cimadom and Tebbich, 2021). Of these, only nests with an intact, undamaged base were considered, as *P. downsi* larvae reside in the lower layer of the nest during the day. *Philornis downsi* abundance (response) was modeled as a function of the volume of dispenser material incorporated into nests (test predictor), including control nests without dispenser material (volume = 0 cm^3^). The model accounted for potential effects of nestling age, nestling number, seasonal effect, and species. A Tweedie model family with “log” link function was applied to fit three separate models. Each model used data from nests treated with dispenser materials from one of the following groups, along with control nests: Cyromazine (treatment N = 43, control N = 79), 1% Permacap (treatment = 47, control = 71), or 0.5% Permacap (treatment = 14, control = 71). In all three models, the full model explained the variation in the data significantly better than the null model, which excluded the test predictor. For the Cyromazine model, the comparison yielded χ^2^ = 10.805, df = 1, p = 0.001; for the 1% Permacap model, χ^2^ = 133.037, df = 1, p < 0.001; and for the 0.5% Permacap model, χ^2^ = 32.771, df = 1, p < 0.001.

#### 2.5.3 Material volume for near-zero *P. downsi* abundance in self-fumigation using Permacap

To quantify the effects of the two Permacap concentrations, we calculated the amount of 1% and 0.5% treated material required to reduce *P. downsi* abundance to near zero. Two separate Tweedie models with a “log” link function were fitted, using *P. downsi* abundance as the response variable. Material volume was included as the test predictor, while nestling age, number of nestlings and the seasonal effect were incorporated as control predictors. Predictions were generated across a range of material volumes, with other variables held constant. For predictions, a near-zero *P. downsi* abundance of 0.01 was used in place of exact zeros, as the model’s “log” link function requires all predicted values to be strictly positive. The respective datasets from the 1% and 0.5% Permacap models, as described in the previous section (effect of self-fumigation on *P. downsi* abundance), were used and continuous variables were not z-transformed. The species identity was excluded for simplification and to focus on the primary relationship between material volume and *P. downsi* abundance.

In the 0.5% Permacap model, the DHARMa outlier test identified seven outliers. Six were nests without treated material and zero *P. downsi* infestations, likely reflecting natural variation. One outlier was a Small Ground-finch nest with treated material (0.12 cm^3^) and high *P. downsi* infestation intensity (69 individuals). This outlier appeared only when species identity was excluded, suggesting species-specific effects. Removing this high-intensity data point eliminated the significance of the outlier test. To evaluate its impact, models were run both with and without this data point. As results remained consistent and biologically plausible, we retained the outlier. Model results are provided in the Supplementary Material for transparency (Supplementary Tables S4, S5). Inclusion of all outliers supports biological variability and did not affect overall conclusions.

#### 2.5.4 Effect of self-fumigation on fledging success

The effect of dispenser material volume (test predictor) on fledging success in nests with hatched nestlings was tested using a binomial response categorized into fledged and failed nests. Nests were classified as fledged if at least one nestling successfully fledged, confirmed by observing fledglings near the nest (Heyer et al., 2021). Only nests where nestling death was not due to predation or nest destruction (e.g. heavy rain) were included. Failed nests with nestlings <7 days old were classified as “dead nestlings” if found empty, assuming parental removal, while nests with nestlings >7 days were classified as “predated” (Cimadom et al., 2014). Analyses were restricted to the three most abundant species in the study area: Small Ground-finch, Small Tree-finch, and Green Warbler-finch. A binomial model family with a “logit” link function was applied to fit three separate models, each corresponding to nests with dispenser material from one of the chemical treatments and control nests that did not contain dispenser material: Cyromazine (treatment N = 33, control N = 58), 1% Permacap (treatment N = 40, control N = 58), and 0.5% Permacap (treatment N = 10, control N = 58). *Philornis downsi* abundance and species identity were used as control predictors. For 0.5% Permacap, insufficient data prevented the calculation of volume effects on fledging success; therefore, the prevalence of nests containing material was used as the test predictor instead, with the same control predictors. Both full models evaluating the effect of Permacap explained a greater proportion of the variation in fledging success compared to the null model, which excluded the test predictor (for the 1% Permacap, χ^2^ = 22.907, df = 1, p < 0.001; for the 0.5% Permacap model, χ^2^ = 5.607, df = 1, p = 0.018). For the Cyromazine model, the comparison did not show a significant difference between full and null model (χ^2^ = 0.023, df = 1, p = 0.881). Additionally, for the 1% Permacap treatment, a separate model was fitted with fledging success as the response variable. The prevalence of nests containing material was included as the binomial test predictor, while *P. downsi* abundance and species identity were included as control predictors. This model explained significantly more variation in the response compared to the null model (χ^2^ = 24.919, df = 1, p < 0.001). Based on the models for both 1% and 0.5% Permacap treatments, with the prevalence of nests containing material as the test predictor, estimated marginal means (EMMs) were calculated with the “emmeans” package to compare predicted fledging probabilities between nests with and without treated material, while accounting for other model predictors.

#### 2.5.5 Effect of the Spritz technique on *P. downsi* abundance

To assess the effect of spraying treatments (test predictor) on *P. downsi* abundance (response), separate Tweedie models with a “log” link function were created for each year, including only undamaged nests with hatched nestlings. The 2023 dataset (N = 92) comprised 13 nests treated with 1% Permacap, 20 with water, and 59 untreated nests. In 2024 (N = 110), 13 nests received 0.5% Permacap, 13 water, and 84 remained untreated. Species identity and the seasonal effect were included as control predictors. The two full models significantly differed from null models without the test predictor (for the 2023 model χ^2^ = 41.046, df = 2, p < 0.001; for the 2024 model χ^2^ = 30.334, df = 2, p < 0.001).

#### 2.5.6 Effect of the Spritz technique on fledging success

The effect of the spraying treatment (test predictor) on fledging success (response) was tested using separate binomial models with a “logit” link function for each study year. In 2023, the dataset (N = 78) included 12 nests treated with 1% Permacap, 17 water-treated nests, and 49 untreated nests. In 2024, the dataset (N = 101) included 13 nests treated with 0.5% Permacap, 12 water-treated nests, and 76 untreated nests. All datasets excluded nests where nestlings died due to factors other than *P. downsi* parasitism (i.e. predation or nest destruction). *Philornis downsi* abundance and species identity were included as control predictors. The full models explained significantly more variation in fledging success than the null models (for the 2023 model χ^2^ = 19.481, df = 2, p < 0.001; for the 2024 model χ^2^ = 21.696, df = 2, p < 0.001).

## 3 Results

### 3.1 Dispenser material use

**Figure 2:**
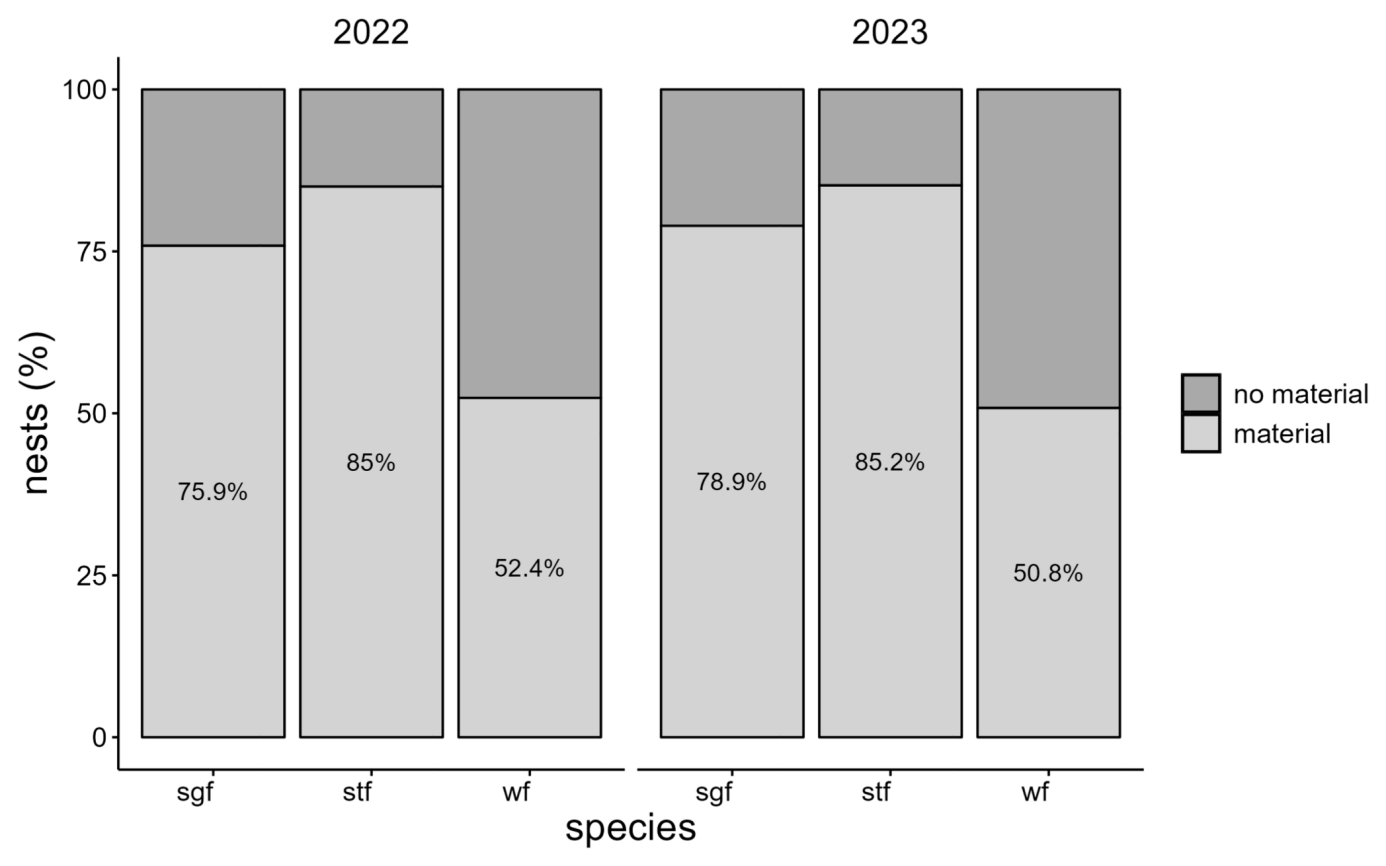
Prevalence of dispenser material (%) in nests of Small Ground-finches (sgf), Small Tree-finches (stf) and Green Warbler-finches (wf) in 2022 and 2023.

**Figure 3:**
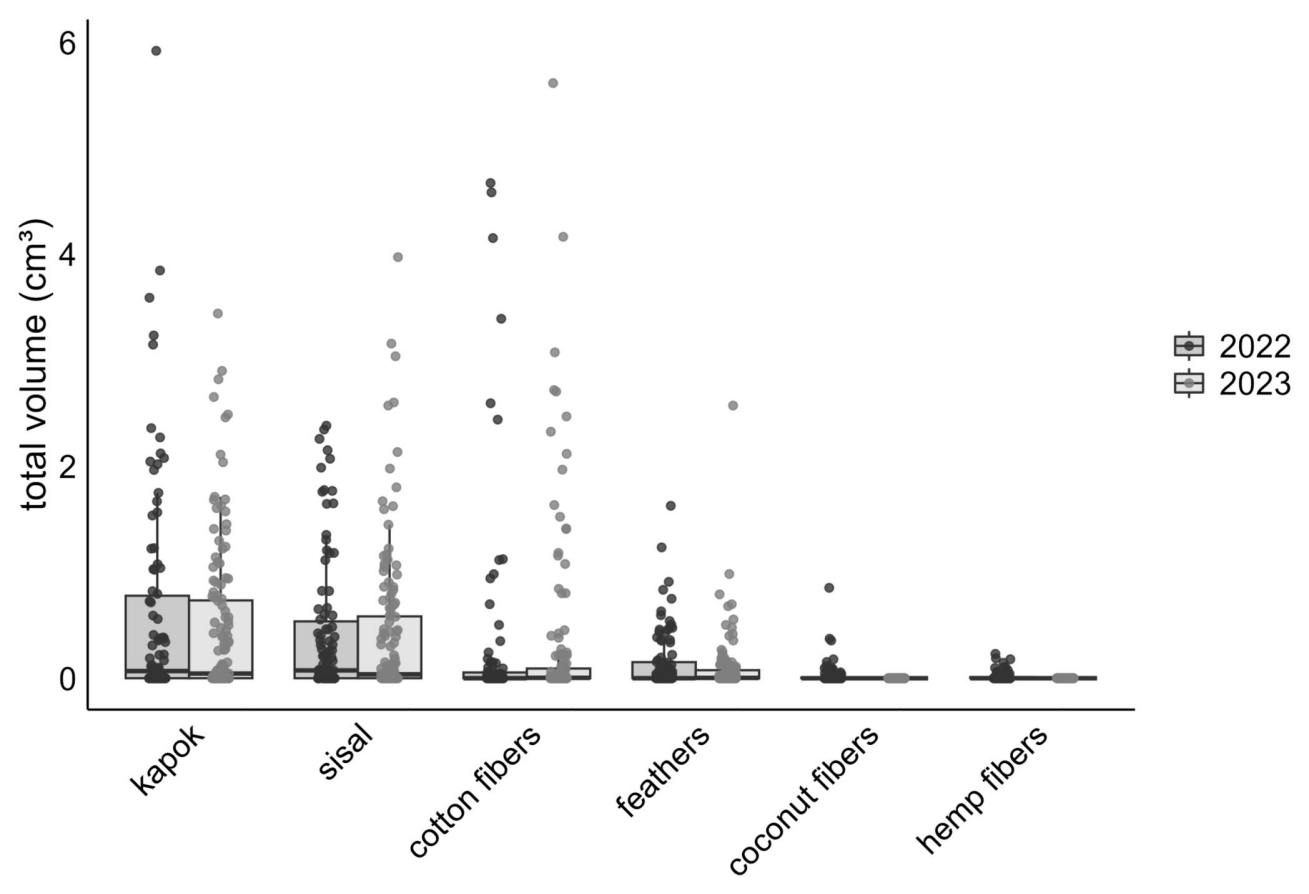
Volume of provided dispenser materials (cm^3^) incorporated in nests across two years, based on data from all nests containing dispenser material of all monitored species: 2022 (light gray) and one for 2023 (dark gray). Error bars indicate the standard error SE of the means.

All monitored species, except the Medium Ground-finch, incorporated dispenser material into their nests. The prevalence of nests containing material and material volume per nest were similar across years (2022: 52.4–85%, 2.04 ± 0.28 cm^3^, N = 71; 2023: 50.8–85.2%, 1.87 ± 0.23 cm^3^, N = 91) and increased significantly over the season. No significant differences in the prevalence of nests containing material were found between year-species combinations (Table 1; Appendix Table A1). Small Tree-finches exhibited the highest prevalence of nests containing material, while Green Warbler-finches had the lowest in both years (Figure 2). Small Tree-finches and Green Warbler-finches incorporated significantly less material by volume than Small Ground-finches (Table 2; Appendix Table A2; Supplementary Material Figure S2). Across species, material preference remained consistent, despite offering six materials in 2022 and only the four most popular in 2023 (Figure 3). Kapok and sisal were favored by all species. Small Ground- and Small Tree-finches also frequently collected cotton fibers, while Green Warbler-finches rarely used this material type (Supplementary Tables S6; S7).

### 3.2 Effect of self-fumigation on *P. downsi* abundance

**Figure 4:**
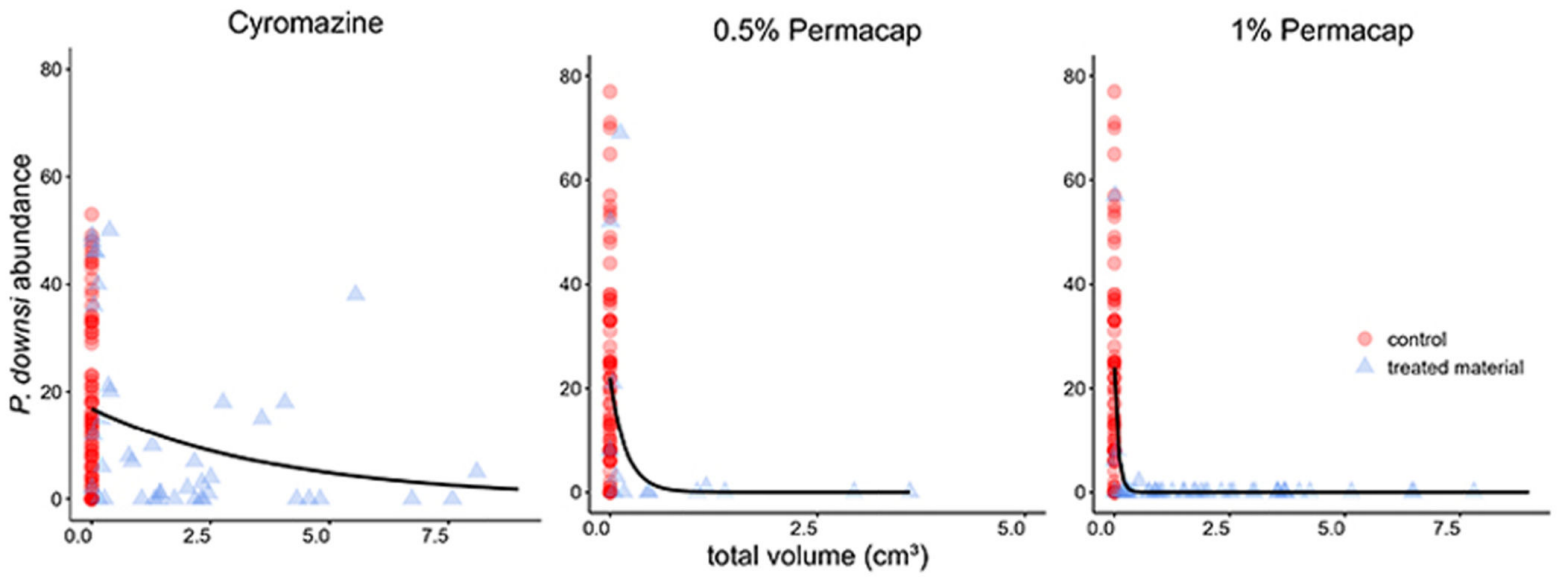
Relationship between total dispenser material volume in nests (including control nests with 0 cm^3^ material volume) and *P. downsi* abundance. The black line represents the predicted *P. downsi* abundance. Panels show the results for different treatments of dispenser material: (left) Cyromazine, (middle) 0.5% Permacap, and (right) 1% Permacap.

In all treatments (Cyromazine, 1% and 0.5% Permacap), higher material volume in nests was significantly associated with lower *P. downsi* abundance. Parasite load increased with nestling number, while no significant seasonal effect was found. Both nestling age and species identity affected parasite abundance only in the Permacap treatments: *P. downsi* abundance increased with nestling age, and Green Warbler-finches had significantly lower parasite loads than Small Ground-finches, while Small Tree-finches did not differ from Small Ground-finches (Tables 3–5; Figure 4).

The volume of 1% Permacap-treated material required to achieve near-zero *P. downsi* abundance across species was 0.6 cm^3^, whereas for 0.5% Permacap it was over twice as high, 1.5 cm^3^ (Supplementary Material Tables S4, S5, S8).

### 3.3 Effect of self-fumigation on fledging success

When material was treated with Cyromazine, material volume had no significant effect on fledging success (Supplementary Material Figure S3; Appendix Table A3). In contrast, for the 1% Permacap treatment, both volume (Table 6) and prevalence of nests with treated material (Appendix Table A4, Supplementary Figure S3) had a significant effect on fledging success in the three most abundant species. The model-based predicted probability of fledging increased from 39.3% (95% CI: 22.3–59.3%) in nests without treated material to 97.6% (95% CI: 88.1–99.5%) in nests with treated material. A similar positive effect was observed for nests containing 0.5% Permacap-treated material (Table 7; Supplementary Figure S3), with predicted fledging success increasing from 48.9% (95% CI: 32.9–65.1%) in untreated nests to 88.9% (95% CI: 57.6–97.9%) in treated nests.

### 3.4 Effect of the Spritz technique on *P. downsi* abundance

**Figure 5:**
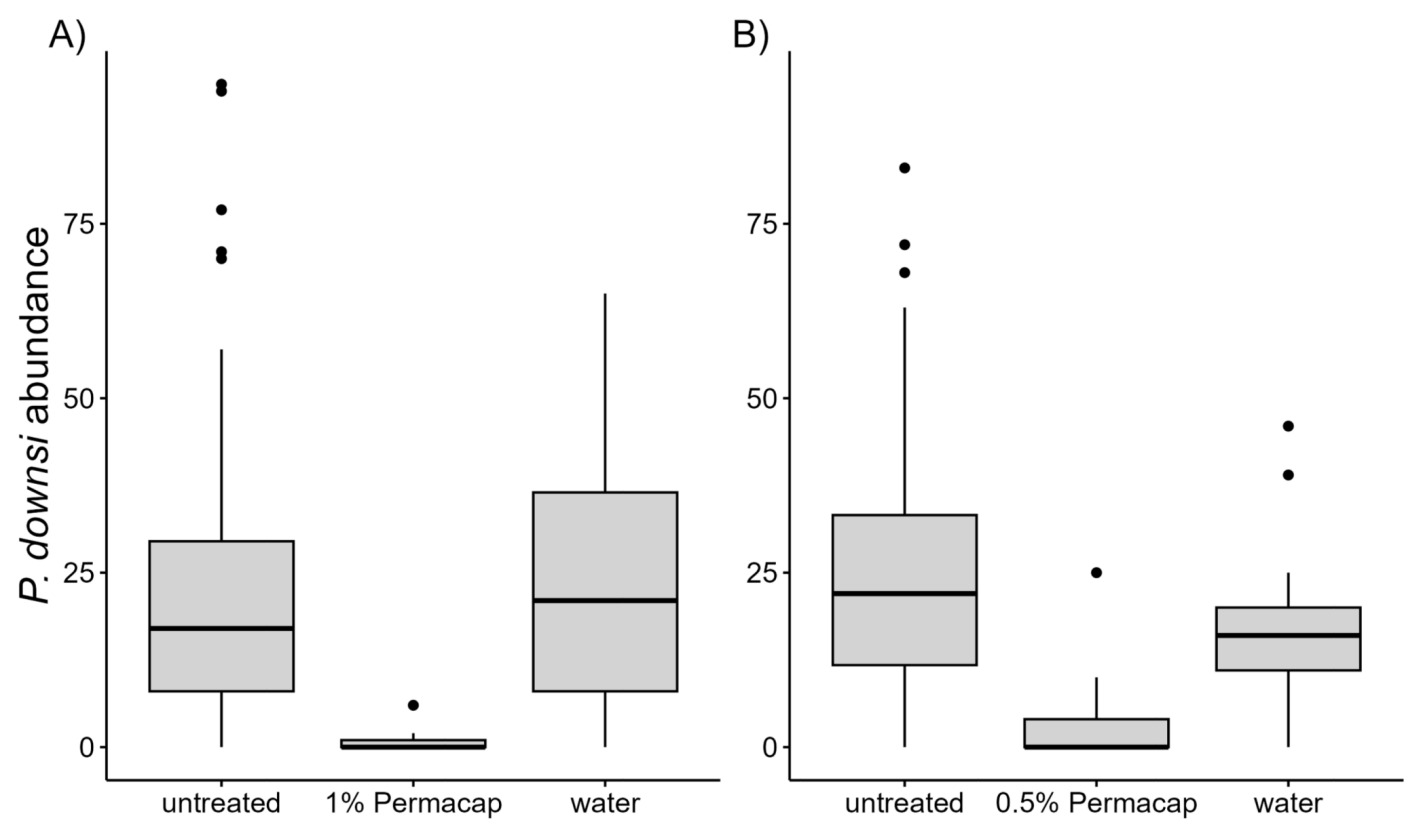
Boxplot showing *P. downsi* abundance by spraying treatment (Permacap and water) in 2023 **(A)** and 2024 **(B)**, with comparisons to untreated nests (no spraying).

Both 1% and 0.5% Permacap treatments resulted in significantly lower *P. downsi* abundance compared to untreated nests, while the water treatment did not differ from untreated nests. No significant seasonal effect on *P. downsi* abundance was detected, indicating that parasite levels were consistent across the study period (Tables 8, 9; Figure 5).

### 3.5 Effect of the Spritz technique on fledging success

**Figure 6:**
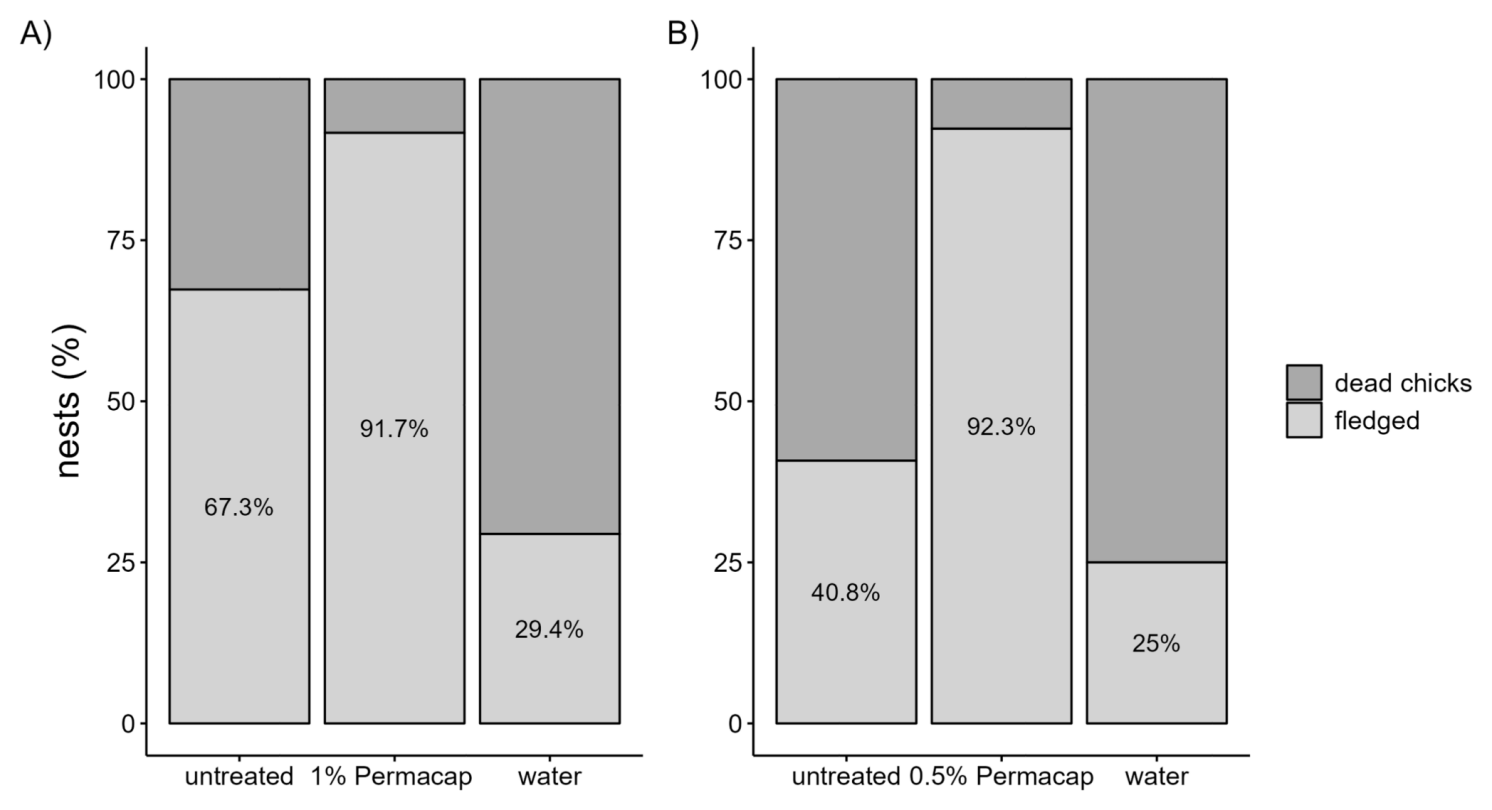
Percentage of successful fledging in Darwin’s finches (including Small Ground-finch, Small Tree-finch, and Green Warbler-finch) based on spraying treatments compared to control nests in 2023 **(A)**, and 2024 **(B)**.

Fledging success was significantly higher in nests treated with 1% and 0.5% Permacap compared to untreated nests. Water treatment negatively affected fledging success in 2023 relative to untreated nests, but no such effect was observed in 2024 (Tables 10, 11; Figure 6).

We observed high nest abandonment in Green Warbler-finches following 1% Permacap treatment (78%), compared to control nests (23%). In contrast, Small Ground-finches and Small Tree-finches showed similar abandonment rates between treated and untreated nests (Small Ground-finch: 25% vs. 23%; Small Tree-finch: 33% vs. 36%). No notable differences in nest abandonment were observed between 0.5% Permacap-treated and untreated nests across all species (Supplementary Material Figure S4).

## 4 Discussion

This study demonstrated the effectiveness of a modified version of the Self-fumigation technique (using Permacap) in reducing *P. downsi* abundance and increasing fledging success across different Darwin’s finch species. Furthermore, the Spritz technique showed measurable improvements in fledging success, indicating its potential as a targeted intervention for critically endangered finch species with specialized nesting behavior and small populations. The findings highlight key challenges in material choice and chemical selection and emphasize the potential for optimizing dosage to balance efficacy and minimize risks, both critical for conservation success.

### 4.1 Material selection and dispenser design

Compared to preliminary studies offering only unprocessed cotton to the bird community in the highlands of Santa Cruz (Causton etal., 2020), our results show that providing a diversity of material types increased acceptance across Darwin’s finch species. Accounting for interspecific differences in material preference, offering kapok, sisal, and cotton fibers enhanced material use, particularly by the targeted Small Tree-finch and Green Warbler-finch.

Birds often modify the inner layer of the nest to protect nestlings from pathogens (Suárez-Rodríguez et al., 2013; Scott-Baumann and Morgan, 2015; Ruiz-Castellano et al., 2018), making the strategic selection of suitable lining materials paramount for optimizing nest-based ectoparasite control via self-fumigation. Nest structure, including material preferences, vary both between species and within species, influenced by factors such as structural characteristics and coloration (Muth et al., 2013; Álvarez et al., 2013; Healy et al., 2015; Ruiz-Castellano et al., 2018), and can also be influenced by species-specific traits, such as body size and bill morphology (Hansell, 2000). Such morphological constraints likely explain the limited use of cotton fibers by the Green Warbler-finch, which is the smallest Darwin’s finch with a slender, insectivorous beak (Hau and Wikelski, 2001). The material selection should be guided by existing knowledge of nest-building behavior, while generally providing a diverse range of materials may be advantageous to accommodate behavioral variability and enhance intervention success.

Installing a single dispenser at a height of 4 m per point with four material types (versus six types and two heights the previous year) did not reduce the proportion of nests containing dispenser material, nor did it significantly decrease the quantity of material used for nest building across the three most abundant Darwin’s finch species at our study area. Optimal Foraging Theory predicts that animals balance resource quality against energy expenditure and predation risk, while Central Place Foraging Theory posits that distance from a central place, such as a nest, influences foraging effort and load size (Stephens and Krebs, 1986; Bell, 1990). Similar principles apply to nest material collection (Hansell, 2000; Mainwaring and Hartley, 2013; Collins et al., 2023). At our study site, all three focal species nested in the forest canopy (Small Tree-finch: mean nest height = 7.1 m, N = 40; Small Ground-finch: mean nest height = 7.1 m, N = 67; Green Warbler-finch: mean nest height = 6.0 m, N = 119; 2023 data). The higher dispenser positioned at 4 m therefore likely matched their primary nesting strata, facilitating access across species.

The same mechanisms likely govern horizontal spatial coverage. With dispensers spaced 50 m apart, 50–85% of nests contained treated material (depending on the species). By comparison, Knutie et al. (2014) reported 85% prevalence of nests containing dispenser material with 40 m spacing along two transects, and Alves et al. (2021) observed 84% prevalence when dispensers were positioned just 4 m from nest boxes. Although dispenser distance was not directly evaluated, nest material use generally declines with distance from the source (Deeming and Mainwaring, 2015; Rydgren et al., 2023; Akresh et al., 2024). Dispenser distribution involves a potential trade-off between logistical feasibility – closer spacing increases deployment effort – and coverage across species, which may be influenced by interspecific differences in material uptake. At our study site, Green Warbler-finches hold the smallest territories (13 m radius) compared to Small Ground-finches (26 m radius) and Small Tree-finches (22 m radius) (calculated from density estimates in Dvorak et al., 2012), which may influence access to dispensers.

Interspecific variation in material volume is likely also constrained by nest size. Among our focal species, Small Ground-finches build the largest nests and Green Warbler-finches the smallest (Kleindorfer and Dudaniec, 2009), matching observed species-specific differences in material volume. Behavioral traits may further contribute to species-specific differences in dispenser material uptake. García-Loor et al. (2025) found that Galapagos landbird species with greater foraging diversity, such as Small Ground-finches, were more exploratory toward novel objects. However, although Common et al. (in prep.) detected species-level differences in material volume per nest, they found no influence of individual variation in neophilia or aggressiveness on material uptake in Darwin’s finches on Floreana Island, Galapagos.

The consistently high prevalence of treated material in nests over two consecutive study years suggests that the dispensers effectively reached a significant portion of the bird populations at this study area thereby providing protection against *P. downsi* parasitism. Although dispensers were used in the same plot over two consecutive years, no increase in the prevalence of nests containing material was observed. The relatively high prevalence of nests with material in 2022, the first year of deployment, may have limited detectable year-to-year differences, potentially due to the finches’ high neophilia (Tebbich et al., 2010). Darwin’s finches likely do not require a prolonged habituation phase to accept new materials. Nevertheless, both the prevalence of nests containing material and the quantity incorporated increased later in the season, suggesting within-season habituation to the dispensers. Consistency in species-specific factors influencing material uptake, such as nest characteristics, territory size, overall preference for the most frequently collected materials, and favored dispenser height, likely explains the lack of substantial differences in material prevalence across species × year combinations, despite changes in dispenser setup.

### 4.2 Type of insecticides and concentration for self-fumigation

Both Permacap and Cyromazine effectively reduced *P. downsi* abundance in nests when used with the Self-fumigation technique. However, only Permacap-impregnated materials improved fledging success, even at the lower 0.5% dosage. Across Small Ground-finches, Small Tree-finches, and Green Warbler-finches, the predicted probability of fledging was 97.6% in nests with treated material, compared to 39.3% in nests without. These results are consistent with previous self-fumigation studies, which reported 95% of nests with at least one fledgling (Knutie et al., 2014) or 95% hatchling survival (Alves et al., 2021) when nests contained dispenser-provided material, and exceed the fledging success observed in earlier Permacap injection studies (Cimadom et al., 2019), which reported 75% fledging success for Small Tree-finches and 78% for Green Warbler-finches (averaged predicted values from nests in a long-term weed management area).

The inability of Cyromazine to improve fledging success in our study is likely due to its failure to completely eliminate larvae, despite substantial incorporation of treated materials into the nest. Cyromazine, primarily used to control nuisance flies in livestock farms (Bueno et al., 2021), shows variable effectiveness depending on dosage and application method, influencing larval survival, fecundity, longevity, and other population parameters (e.g. Friedel and McDonell, 1985; Donahue et al., 2017; Khan, 2023). Similarly, Causton et al. (2019, 2020) reported varying results with different Cyromazine concentrations and application methods. Spraying 5 mL of 0.2 g/L moderately improved bird reproductive success and slightly reduced fly emergence by suppressing larval activity, although total parasite numbers were not significantly reduced. Injection treatments with the same dosage showed no significant effect (Causton et al., 2019). Using a higher concentration, spraying ~8 mL of 0.4 g/L inside nests, including under the lining, significantly reduced *P. downsi* abundance, resulting in an 85% fledging success rate (Causton et al., 2020). These findings suggest that for Cyromazine to effectively increase fledging success, a thorough treatment of the entire nest chamber may be necessary, as demonstrated by Causton et al. (2020). Further research is necessary to improve the performance of Cyromazine when used with the Self-fumigation technique, possibly by combining it with a surfactant or another growth inhibitor.

In contrast, minimal amounts of treated material were required in the nests to fully reduce *P. downsi* abundance when material was treated with 1% and 0.5% Permacap. Specifically, our results showed that reducing *P. downsi* abundance to near-zero required a minimum of 1.5 cm^3^ of 0.5% Permacap-treated material, compared to only 0.6 cm^3^ with 1% Permacap. To contextualize our findings, Knutie et al. (2014) reported that approximately 1 g of cotton treated with a 1% permethrin solution was needed to reduce *P. downsi* infestation toward zero. Assuming a cotton density of 0.47 g/cm^3^ (National Cotton Council of America, 2025), the 0.6 cm^3^ of material used in our 1% Permacap treatment corresponds to approximately 0.3 g. This discrepancy may be attributed to the use of the controlled-release permethrin formulation (Permacap) in our study, which likely prolonged insecticidal efficacy and thus reduced the amount of material required.

Small Ground-finches exceeded the 1.5 cm^3^ threshold of 0.5% Permacap-treated material that was associated with near-zero *P. downsi* abundance. Small Tree-finches did so only in 2022, while Green Warbler-finches remained below the threshold in both years. Nonetheless, both species showed improved fledging success, suggesting that full parasite elimination may not be required. However, identifying a specific *P. downsi* abundance threshold associated with fledging success was not possible, primarily due to the natural increase in infestation levels with nestling age. Additionally, the high effectiveness of Permacap in reducing *P. downsi* abundance resulted in minimal variation in the data. Nevertheless, given that there are species-specific differences in material collection, using the 0.5% Permacap concentration could pose a potential risk for unbalanced conservation effects, disproportionately benefiting species that collect more material, such as Small Ground-finches. To ensure effective self-fumigation across different species and achieve a balanced conservation outcome, additional research is needed on the attractiveness of materials for target species.

### 4.3 Insecticide concentration in the Spritz-technique

The newly developed spraying device made the Spritz technique easily applicable and the tested method proved effective for controlling *P. downsi* in target nests. Both 1% and 0.5% Permacap solutions significantly reduced *P. downsi* abundance and increased fledging success compared to water-treated and untreated nests. However, the abandonment rate was significantly higher in nests sprayed with 1% Permacap, particularly in Green Warbler-finches (7 out of 9 nests were abandoned). This sensitivity, potentially attributed to the odor of Permacap, was previously observed in this species. Cimadom et al. (2019) reported nest abandonment in Green Warbler-finches following Permectrin^™^ II treatment, an effect mitigated by the use of 0.5% Permacap. By halving the dose to 0.5% Permacap in the present study, we were able to prevent nest abandonment while still improving fledging success in treated nests, suggesting that further testing of even lower concentrations may help refine the balance between efficacy and safety.

## 5 Conclusion

The high survival rates of nestlings achieved in our study using the modified Self-fumigation technique and the Spritz technique demonstrate their potential as stop-gap measures to address the threat of *P. downsi* to Galapagos’ landbird species. In addition to being effective, these methods – particularly the Self-fumigation technique – considerably reduce the effort required by conservation workers to treat nests and enable a greater number of nests to be treated. Moreover, the study shows how the Permacap concentration in both methods can be fine-tuned to maintain efficacy while minimizing insecticide concentration. These highly efficient and easy-to-use tools are now available for immediate *P. downsi* control, and are expected to enhance fledging success in threatened Darwin’s finch species, potentially contributing to ongoing conservation efforts to support population recovery. Additionally, the use of these tools in re-wilding programs, contemplated for islands in Galapagos where invasive rats have been eradicated, will help restore bird populations that have gone extinct.

Lastly, while the current system has proven highly successful, its broader applicability in Galapagos and other parts of the world will depend on the characteristics of the focal species and their locations. Bird material specificity, nest architecture, dispenser distance and height, and the potential effects of insecticide concentrations on target ectoparasites must be carefully evaluated. Preliminary trials and targeted field observations will be crucial for adapting these methods to other bird host-parasite systems, ensuring conservation outcomes are optimized across diverse ecological settings.

## Supplementary Material

The Supplementary Material for this article can be found online at:  https://www.frontiersin.org/articles/10.3389/fcosc.2025.1591266/full#supplementary-material

Supplementary Material

Supplementary Tables

## Figures and Tables

**Table 1:** GLM results: material prevalence by year and species; reference categories are Small Ground-finch (sgf) for species and 2022 for year; stf = Small Tree-finch, wf = Green Warbler-finch; N = 217; asterisks indicate significance (*p < 0.05, ***p < 0.001).

Predictors	Estimate (95% CI)	SE	Z-value	P-value
(Intercept)	-1.325 (-2.612, -0.039)	0.657	-2.019	0.044 *
year [2023]	0.753 (-0.478, 1.985)	0.628	1.199	0.231
species [stf]	0.775 (-0.793, 2.342)	0.800	0.969	0.333
species [wf]	-0.793 (-1.938, 0.351)	0.584	-1.358	0.174
seasonal effect	0.035 (0.021, 0.048)	0.007	5.076	<0.001 ***
year [2023] × species [stf]	-0.171 (-2.240, 1.899)	1.056	-0.161	0.872
year [2023] × species [wf]	-0.238 (-1.740, 1.264)	0.766	-0.311	0.756

**Table 2:** GLM results: dispenser material volume (cm^3^) by year and species; reference categories are Small Ground-finch (sgf) for species and 2022 for year; stf = Small Tree-finch, wf = Green Warbler-finch; N = 145; asterisks indicate significance (***p < 0.001).

Predictors	Estimate (95% CI)	SE	Z-value	P-value
(Intercept)	0.953 (0.522, 1.385)	0.220	4.329	<0.001 ***
year [2023]	0.309 (-0.014, 0.632)	0.165	1.873	0.061
species [stf]	-0.604 (-0.962, -0.247)	0.182	-3.315	<0.001 ***
species [wf]	-0.933 (-1.266, -0.600)	0.170	-5.487	<0.001 ***
seasonal effect	0.009 (0.005, 0.014)	0.002	4.000	<0.001 ***
year [2023] × species [stf]	-0.229 (-0.700, 0.241)	0.240	-0.956	0.339
year [2023] × species [wf]	-0.395 (-0.832, 0.042)	0.223	-1.771	0.077

**Table 3:** GLM results, effect of volume (cm^3^) of dispenser material with Cyromazine treatment on *P. downsi* abundance; reference category is Small Ground-finch (sgf) for species; stf = Small Tree-finch, wf = Green Warbler-finch; N = 122; asterisks indicate significance (*p < 0.05, **p < 0.01, ***p < 0.001).

Predictors	Estimate (95% CI)	SE	Z-value	P-value
(Intercept)	2.608 (2.20, 3.02)	0.210	12.408	< 0.001 ***
z.volume	-0.428 (-0.69, -0.16)	0.134	-3.182	0.001 **
z.nestling age	0.046 (-0.14, 0.23)	0.094	0.489	0.625
z.seasonal effect	-0.120 (-0.32, 0.08)	0.103	-1.165	0.244
z.nestling number	0.273 (0.08, 0.47)	0.100	2.730	0.006 **
species [stf]	0.351 (-0.15, 0.85)	0.255	1.376	0.169
species [wf]	-0.271 (-0.82, 0.27)	0.278	-0.976	0.329

**Table 4:** GLM results, effect of volume (cm^3^) of dispenser material with 1% Permacap treatment on *P. downsi* abundance; reference category is Small Ground-finch (sgf) for species; stf = Small Tree-finch, wf = Green Warbler-finch; N = 118; asterisks indicate significance (*p < 0.05,**p < 0.01, ***p < 0.001).

Predictors	Estimate (95% CI)	SE	Z-value	P-value
(Intercept)	-6.827 (-10.80, -2.86)	2.025	-3.372	0.001 ***
z.volume	-21.194 (-29.33, -13.06)	4.151	-5.105	< 0.001 ***
z.nestling age	0.302 (0.13, 0.48)	0.089	3.399	0.001 **
z.nestling number	0.254 (0.06, 0.45)	0.100	2.541	0.011 *
z.seasonal effect	-0.013 (-0.20, 0.17)	0.093	-0.144	0.886
species [stf]	0.124 (-0.37, 0.62)	0.253	0.491	0.623
species [wf]	-0.690 (-1.11, -0.27)	0.216	-3.202	0.001 **

**Table 5:** GLM results, effect of volume (cm^3^) of dispenser material with 0.5% Permacap treatment on *P. downsi* abundance; reference category is Small Ground-finch (sgf) for species; stf = Small Tree-finch, wf = Green Warbler-finch; N = 85; asterisks indicate significance (*p < 0.05, **p < 0.01, ***p < 0.001).

Predictors	Estimate (95% CI)	SE	Z-value	P-value
(Intercept)	2.931 (2.492, 3.371)	0.224	13.074	< 0.001 ***
z.volume	-2.555 (-3.986, -1.123)	0.730	-3.498	0.001 **
z.nestling_age	0.276 (0.089, 0.462)	0.095	2.901	0.004 **
z.nestling_number	0.207 (0.022, 0.392)	0.094	2.193	0.028 *
z.seasonal_effect	-0.022 (-0.208, 0.165)	0.095	-0.229	0.819
species [stf]	-0.095 (-0.599, 0.409)	0.257	-0.370	0.711
species [wf]	-0.805 (-1.223, -0.387)	0.213	-3.773	< 0.001 ***

**Table 6:** GLM results, effect of volume (cm^3^) of dispenser material with 1% Permacap treatment on fledging success; reference category is Small Ground-finch (sgf) for species; stf = Small Tree-finch, wf = Green Warbler-finch; N = 98; asterisks indicate significance (** p < 0.01).

Predictors	Estimate (95% CI)	SE	Z-value	P-value
(Intercept)	1.802 (−0.263, 3.867)	1.054	1.710	0.087
z.volume	6.517 (1.637, 11.398)	2.490	2.617	0.009 **
z. *P. downsi *abundance	0.590 (−0.057, 1.237)	0.330	1.787	0.074
species [sgf]	1.579 (−0.205, 3.363)	0.910	1.735	0.083
species [wf]	2.378 (0.720, 4.036)	0.846	2.811	0.005 **

**Table 7:** GLM results, effect of 1% Permacap spraying treatment on *P. downsi* abundance (year 2023); reference categories are untreated nests (no spraying) for spray and Small Ground-finch (sgf) for species; stf = Small Tree-finch, wf = Green Warbler-finch; N = 92; asterisks indicate significance (**p < 0.01, ***p < 0.001).

Predictors	Estimate (95% CI)	SE	Z-value	P-value
(Intercept)	−1.072 (−2.517, 0.374)	0.738	-1.453	0.146
material [TRUE]	2.121 (0.141, 4.100)	1.010	2.099	0.036 *
z.P. *downsi *abundance	0.582 (–0.076, 1.240)	0.336	1.735	0.083
species [sgf]	1.384 (–0.333, 3.101)	0.876	1.580	0.114
species [wf]	1.696 (0.091, 3.301)	0.819	2.072	0.038 *

**Table 8:** GLM results, effect of dispenser material prevalence (0.5% Permacap treatment) on fledging success; reference category is Small Ground-finch (sgf) for species; stf = Small Tree-finch, wf = Green Warbler-finch; N = 63; asterisks indicate significance (*p < 0.05).

Predictors	Estimate (95% CI)	SE	Z-value	P-value
(Intercept)	3.530 (3.204, 3.855)	0.166	21.255	<0.001 ***
spray [1% PC]	-3.432 (-4.682, -2.182)	0.638	-5.381	<0.001 ***
spray [water]	-0.017 (-0.442, 0.407)	0.217	-0.080	0.936
z.seasonal effect	0.082 (-0.117, 0.280)	0.101	0.804	0.421
species [stf]	-0.312 (-0.859, 0.235)	0.279	-1.119	0.263
species [wf]	-0.669 (-1.089, -0.249)	0.214	-3.123	0.002 **

**Table 9:** GLM results, effect 0.5% Permacap spraying treatment on *P. downsi* abundance (year 2024); reference categories are untreated nests (no spraying) for spray and Small Ground-finch (sgf) for species; stf = Small Tree-finch, wf = Green Warbler-finch; N = 110; asterisks indicate significance (*p < 0.05, ***p < 0.001).

Predictors	Estimate (95% CI)	SE	Z-value	P-value
(Intercept)	3.578 (3.206, 3.950)	0.190	18.841	<0.001 ***
spray [0.5% PC]	-1.841 (-2.567, -1.115)	0.370	-4.972	<0.001 ***
spray [water]	-0.413 (-0.871, 0.046)	0.234	-1.765	0.078
z.seasonal effect	-0.114 (-0.251, 0.023)	0.070	-1.634	0.102
species [stf]	-0.110 (-0.611, 0.392)	0.256	-0.429	0.668
species [wf]	-0.490 (-0.899, -0.080)	0.209	-2.344	0.019 *

**Table 10:** GLM results, effect spraying treatment on fledging success (year 2023); reference categories are untreated nests (no spraying) for spray and Small Ground-finch (sgf) for species; stf = Small Tree-finch, wf = Green Warbler-finch; N = 78; asterisks indicate significance (*p < 0.05).

Predictors	Estimate (95% CI)	SE	Z-value	P-value
(Intercept)	0.462 (-0.619, 1.543)	0.552	0.838	0.402
spray [1% PC]	2.931 (0.561, 5.302)	1.209	2.424	0.015 *
spray [water]	-1.677 (-3.012, -0.341)	0.681	-2.461	0.014 *
z.P. *downsi *abundance	0.933 (0.174, 1.691)	0.387	2.409	0.016 *
species [stf]	-0.475 (-2.182, 1.231)	0.871	-0.546	0.585
species [wf]	0.515 (-0.778, 1.807)	0.660	0.780	0.435

**Table 11:** GLM results, effect spraying treatment on fledging success (year 2024); reference categories are untreated nests (no spraying) for spray and Small Ground-finch (sgf) for species; stf = Small Tree-finch, wf = Green Warbler-finch; N = 101; asterisks indicate significance (**p < 0.01).

Predictors	Estimate (95% CI)	SE	Z-value	P-value
(Intercept)	-1.300 (-2.850, 0.250)	0.790	-1.649	0.099
spray [0.5% PC]	4.170 (1.630, 6.710)	1.300	3.219	0.001 **
spray [water]	-0.360 (-1.850, 1.140)	0.760	-0.474	0.639
z.P. *downsi *abundance	0.410 (-0.100, 0.920)	0.260	1.585	0.113
species [stf]	-0.820 (-2.930, 1.300)	1.080	-0.759	0.448
species [wf]	1.230 (-0.410, 2.860)	0.830	1.482	0.141

## Data Availability

The datasets presented in this study can be found in online repositories. The names of the repository/repositories and accession number(s) can be found below: https://phaidra.univie.ac.at/o:2120691; https://phaidra.univie.ac.at/o:2120692.
