# Supplementary material for: Innovations in invasive parasite control: enhancing nest treatment techniques to combat the threat of the avian vampire fly Philornis downsi in Galapagos
Source: Front Conserv Sci. Author manuscript; Available in PMC 2026 Jan 6. (PMC7618593; doi:10.3389/fcosc.2025.1591266)
Supplement: Supplementary Material [file EMS211895-supplement-Supplementary_Material.docx]

**Supplementary Material**

Tab. S1: Quantity of material types used to fill one dispenser in 2022 and 2023. A dispenser was considered as complete when the contained material was estimated to have a total weight of 40 g in 2022 and 45 g in 2023. These indications had to be followed when refilling a dispenser, as the volume of sprayed insecticide solution was dependent on the weight of the material.

| **year** | **material type** | **weight (g)** |
| --- | --- | --- |
| 2022 | sisal fibers | 6 |
|  | cotton fibers | 14 |
|  | chicken feathers | 4 |
|  | kapok | 4 |
|  | coconut fibers | 6 |
|  | hemp fibers | 6 |
| 2023 | sisal fibers | 15 |
|  | cotton fibers | 15 |
|  | chicken feathers | 10 |
|  | kapok | 5 |

*Tab. S2. Amount of material refilled in dispensers of different heights in 2022 and 2023.*

| **weight (g)** | | | | | | | | |
| --- | --- | --- | --- | --- | --- | --- | --- | --- |
|  | **dispenser height** | **kapok** | **sisal** | **cotton fibers** | **feathers** | **coconut fibers** | **hemp fibers** | **total** |
| 2022 | 1.5 m | 16 | 106 | 55 | 15 | 7 | 33 | 232 |
|  | 4 m | 37 | 156 | 117 | 39 | 16 | 53 | 418 |
| 2023 | 4 m | 74 | 336 | 241 | 46 | - | - | 697 |
| **volume (cm^3^)** | | | | | | | | |
|  | **dispenser height** | **kapok** | **sisal** | **cotton fibers** | **feathers** | **coconut fibers** | **hemp fibers** | **total** |
| 2022 | 1.5 m | 53 | 73 | 36 | 22 | 6 | 27 | 217 |
|  | 4 m | 123 | 108 | 76 | 57 | 14 | 44 | 422 |
| 2023 | 4 m | 247 | 232 | 156 | 68 | - | - | 703 |

Tab. S3 Specific density (g/ cm³) and volume (cm³/ g) of dispenser material types used for calculations.

| **material type** | **density (g/ cm^3^)** | **volume (cm³/ g)** |
| --- | --- | --- |
| sisal fibers | 1.45 | 0.69 |
| cotton fibers | 1.54 | 0.65 |
| kapok | 0.3 | 3.33 |
| chicken feathers | 0.68 | 1.47 |
| coconut fibers | 1.15 | 0.87 |
| hemp fibers | 1.2 | 0.83 |

Tab. S4: GLM results showing the effect of dispenser material volume (cm³) treated with 0.5% Permacap on *P. downsi* abundance, used to predict the material quantity required for near-zero infestation; N = 85; asterisks indicate significance (* p < 0.05, ** p < 0.01, *** p < 0.001).

| **predictor** | **estimate (95% CI)** | **SE** | **z-value** | **p-value** |
| --- | --- | --- | --- | --- |
| (Intercept) | 1.590 (0.837, 2.342) | 0.384 | 4.142 | <0.001 *** |
| volume | -4.115 (-6.647, -1.584) | 1.292 | -3.186 | 0.001 ** |
| nestling age | 0.046 (0.004, 0.088) | 0.021 | 2.163 | 0.031 * |
| nestling number | 0.467 (0.197, 0.738) | 0.138 | 3.385 | <0.001 *** |
| seasonal effect | 0.003 (-0.005, 0.011) | 0.004 | 0.746 | 0.456 |

Tab. S5: GLM results showing the effect of dispenser material volume (cm³) treated with 0.5% Permacap on *P. downsi* abundance, used to predict the material quantity required for near-zero infestation (excluding one outlier); N=84; asterisks indicate significance (*** p < 0.001).

| **predictors** | **estimate (95% CI)** | **SE** | **z-value** | **p-value** |
| --- | --- | --- | --- | --- |
| (Intercept) | 1.389 (0.658, 2.120) | 0.373 | 3.723 | <0.001 *** |
| volume | -5.211 (−7.997, −2.425) | 1.422 | -3.666 | <0.001 *** |
| nestling age | 0.052 (0.012, 0.092) | 0.020 | 2.558 | 0.011 * |
| nestling number | 0.459 (0.203, 0.716) | 0.131 | 3.509 | <0.001 *** |
| seasonal effect | 0.005 (−0.002, 0.013) | 0.004 | 1.403 | 0.161 |

Tab. S6: Average volume of dispenser materials (cm³, mean ± SE) incorporated into nests (sgf = Small Ground-finch, stf = Small Tree-finch, wf = Green Warbler-finch, wpf = Woodpecker Finch, ltf = Large Tree-finch) and years (2022, 2023).

| **species** | **year** | **kapok** | **sisal** | **cotton fibers** | **feathers** | **coconut fibers** | **hemp fibers** | **N** |
| --- | --- | --- | --- | --- | --- | --- | --- | --- |
| sgf | 2022 | 1.15 ± 0.21 | 0.9 ± 0.17 | 0.82 ± 0.32 | 0.32 ± 0.19 | 0.07 ± 0.07 | 0.03 ± 0.06 | 22 |
| sgf | 2023 | 1.2 ± 0.15 | 1.21 ± 0.19 | 1.02 ± 0.25 | 0.27 ± 0.49 |  |  | 30 |
| stf | 2022 | 0.97 ± 0.38 | 0.42 ± 0.17 | 0.39 ± 0.25 | 0.1 ± 0.02 | 0.01 ± 0.01 | 0.02 ± 0.06 | 17 |
| stf | 2023 | 0.76 ± 0.17 | 0.36 ± 0.10 | 0.04 ± 0.10 | 0.05 ± 0.11 |  |  | 23 |
| wf | 2022 | 0.2 ± 0.07 | 0.32 ± 0.09 | 0.02 ± 0.01 | 0.21 ± 0.03 | 0.02 ± 0.03 | 0.02 ± 0.03 | 22 |
| wf | 2023 | 0.1 ± 0.03 | 0.16 ± 0.05 | 0.06 ± 0.15 | 0.04 ± 0.06 |  |  | 31 |
| wpf | 2022 | 2.68 ± 1.37 | 2.25 ± 0.99 | 0.18 ± 0.18 | 0.32 ± 0.12 | 0.17 ± 0.09 | 0.07 ± 0.05 | 3 |
| wpf | 2023 | 0.05 ± 0.05 | 0.04 ± 0.04 | 0.63 ± 0.60 | 0.06 ± 0.06 |  |  | 2 |
| ltf | 2022 | 0.36 | 0.21 | 0.18 |  |  | 0.03 | 1 |
| ltf | 2023 | 1.22 | 0.98 | 0.03 | 0.03 |  |  | 1 |

Tab. S7: Average mass of dispenser materials (g, mean ± SE) incorporated into nests (sgf = Small Ground-finch, stf = Small Tree-finch, wf = Green Warbler-finch, wpf = Woodpecker Finch, ltf = Large Tree-finch) and years (2022, 2023).

| **species** | **year** | **kapok** | **sisal** | **cotton fibers** | **feathers** | **coconut fibers** | **hemp fibers** | **N** |
| --- | --- | --- | --- | --- | --- | --- | --- | --- |
| sgf | 2022 | 1.78 ± 0.33 | 1.39 ± 0.26 | 1.27 ± 0.50 | 0.49 ± 0.13 | 0.11 ± 0.06 | 0.05 ± 0.02 | 22 |
| sgf | 2023 | 1.84 ± 0.23 | 1.86 ± 0.30 | 1.58 ± 0.38 | 0.41 ± 0.14 |  |  | 30 |
| stf | 2022 | 1.50 ± 0.59 | 0.64 ± 0.27 | 0.60 ± 0.39 | 0.15 ± 0.06 | 0.01 ± 0.01 | 0.04 ± 0.02 | 17 |
| stf | 2023 | 1.17 ± 0.26 | 0.56 ± 0.15 | 0.06 ± 0.03 | 0.08 ± 0.04 |  |  | 23 |
| wf | 2022 | 0.30 ± 0.10 | 0.50 ± 0.14 | 0.03 ± 0.01 | 0.32 ± 0.11 | 0.02 ± 0.01 | 0.03 ± 0.01 | 22 |
| wf | 2023 | 0.15 ± 0.05 | 0.25 ± 0.07 | 0.09 ± 0.04 | 0.06 ± 0.02 |  |  | 31 |
| wpf | 2022 | 2.68 ± 1.37 | 2.25 ± 0.99 | 0.18 ± 0.18 | 0.32 ± 0.12 | 0.17 ± 0.09 | 0.07 ± 0.05 | 3 |
| wpf | 2023 | 0.05 ± 0.05 | 0.04 ± 0.04 | 0.63 ± 0.60 | 0.06 ± 0.06 |  |  | 2 |
| ltf | 2022 | 0.55 | 0.32 | 0.28 |  |  | 0.05 | 1 |
| ltf | 2023 | 1.88 | 1.51 | 0.04 | 0.04 |  |  | 1 |

Tab. S8: GLM results showing the effect of dispenser material volume (cm³) treated with 1% Permacap on *P. downsi* abundance, used to predict the material quantity required for near-zero infestation; N = 118; asterisks indicate significance (** p < 0.01, *** p < 0.001).

| **predictors** | **estimate (95% CI)** | **SE** | **z-value** | **p-value** |
| --- | --- | --- | --- | --- |
| (Intercept) | 1.395 (0.64, 2.15) | 0.385 | 3.622 | < 0.001 *** |
| volume | -11.843 (-16.17, -7.52) | 2.208 | -5.364 | < 0.001 *** |
| nestling age | 0.057 (0.02, 0.10) | 0.021 | 2.682 | 0.007 ** |
| nestling number | 0.474 (0.22, 0.73) | 0.130 | 3.636 | < 0.001 *** |
| seasonal effect | 0.004 (-0.004, 0.01) | 0.004 | 0.951 | 0.341 |

Tab. S9: Overview of all GLMs used in the analysis, including model type, response and predictor variables, distribution family and link function, sample size (N), and notes on the data subset included. Species abbreviations: Small Ground-finches (sgf), Small Tree-finches (stf), and Green Warbler-finches (wf).

| **model** | **model type** | **response variable** | **test predictor** | **control predictors** | **random effects** | **distri-bution** | **N** | **notes** |
| --- | --- | --- | --- | --- | --- | --- | --- | --- |
| self-fumigation material use (a) | GLM | prevalence of dispenser materials (1/0) | year × species interaction | seasonal effect | - | binomial (link = logit) | 217 | complete nests from sgf, stf, wf included |
| self-fumigation material use (b) | GLM | volume (cm³) of dispenser material | year × species interaction | seasonal effect | - | gaussian  (link = identity) | 145 | complete nests that contained dispenser material from sgf, stf, wf included |
| self fumigation - *P. downsi* abundance (a) | GLM | *P. downsi* abundance | volume (cm³) of dispenser material treated with Cyromazine, | nestling age, nestling number, seasonal effect, species | - | tweedie (link = log) | 122  (Cyromazine treatment = 43, control = 79) | complete undamaged nests with hatched nestlings from sgf, stf, wf, study year 2022 |
| self fumigation - *P. downsi* abundance (b) | GLM | *P. downsi* abundance | volume (cm³) of dispenser material treated with 1% Permacap | nestling age, nestling number, seasonal effect, species | - | tweedie (link = log) | 118 (1% Permacap treatment = 47, control = 71) | complete undamaged nests with hatched nestlings from sgf, stf, wf, study year 2023 |
| self fumigation - *P. downsi* abundance (c) | GLM | *P. downsi* abundance | volume (cm³) of dispenser material treated with 0.5% Permacap | nestling age, nestling number, seasonal effect, species | - | tweedie (link = log) | 85 (0.5% Permacap treatment = 14, control = 71) | complete undamaged nests with hatched nestlings from sgf, stf, wf, study year 2023 |
| self fumigation - *P. downsi* abundance (b.1) | GLM | *P. downsi* abundance | volume (cm³) of dispenser material treated with 1% Permacap | nestling age, nestling number, seasonal effect | - | tweedie (link = log) | 118 (1% Permacap treatment = 47, control = 71) | complete undamaged nests with hatched nestlings from sgf, stf, wf, study year 2023 -> used to estimate 1% Permacap volume needed for near-zero infestations |
| self fumigation - *P. downsi* abundance (c.1) | GLM | *P. downsi* abundance | volume (cm³) of dispenser material treated with 0.5% Permacap | nestling age, nestling number, seasonal effect | - | tweedie (link = log) | 85 (0.5% Permacap treatment = 14, control = 71) | complete undamaged nests with hatched nestlings from sgf, stf, wf, study year 2023  -> used to estimate 0.5% Permacap volume needed for near-zero infestations |
| self fumigation - fledging success (a) | GLM | fledging success (1/0) | volume (cm³) of dispenser material treated with Cyromazine | *P. downsi* abundance, species | - | binomial (link = logit) | 91  (Cyromazine treatment = 33, control = 58) | only nests with hatched nestlings without predation/destruction included, from sgf, stf, wf, study year 2022 |
| self fumigation - fledging success (b) | GLM | fledging success (1/0) | volume (cm³) of dispenser material treated with 1% Permacap | *P. downsi* abundance, species | - | binomial (link = logit) | 98 (1% Permacap treatment = 40, control = 58) | only nests with hatched nestlings without predation/destruction included, from sgf, stf, wf, study year 2023 |
| self fumigation - fledging success (c) | GLM | fledging success (1/0) | material prevalence (1/0) of dispenser material treated with 0.5% Permacap | *P. downsi* abundance, species | - | binomial (link = logit) | 68 (0.5% Permacap treatment = 10, control = 58) | only nests wit hatched nestlings without predation/destruction included, from sgf, stf, wf, study year 2023 |
| self fumigation - fledging success (b.1) | GLM | fledging success (1/0) | material prevalence (1/0) of dispenser material treated with 0.5% Permacap | *P. downsi* abundance, species | - | binomial (link = logit) | 98 (1% Permacap treatment = 40, control = 58) | only nests with hatched nestlings without predation/destruction included, from sgf, stf, wf, study year 2023, model used for estimating group-level differences in fledging probabilities |
| Spritz –technique – *P. downsi* abundance (a) | GLM | *P. downsi* abundance | treatment (1% Permacap, water, untreated) | species, seasonal effect | - | tweedie (link = log) | 92 (1% Permacap = 13, water = 20, untreated = 59) | only undamaged nests with hatched nestlings included, study year 2023 |
| Spritz –technique – *P. downsi* abundance (b) | GLM | *P. downsi* abundance | treatment  (0.5% Permacap, water, untreated) | species identity, seasonal effect | - | tweedie (link = log) | 110 (0.5% Permacap 13, water = 13, untreated = 84) | only undamaged nests with hatched nestlings included, study year 2024 |
| Spritz –technique – fledging success (a) | GLM | fledging success (1/0) | treatment  (1% Permacap, water, untreated) | *P. downsi* abundance species | - | binomial (link = logit) | 78 (1% Permacap = 12, water = 17, untreated = 49) | only nests with hatched nestlings without predation/destruction included, study year 2023 |
| Spritz –technique – fledging success (b) | GLM | fledging success (1/0) | treatment  (0.5% Permacap, water, untreated) | *P. downsi* abundance species | - | binomial (link = logit) | 101 (0.5% Permaca2 13, water = 13, untreated = 76) | only nests with hatched nestlings without predation/destruction included, study year 2024 |


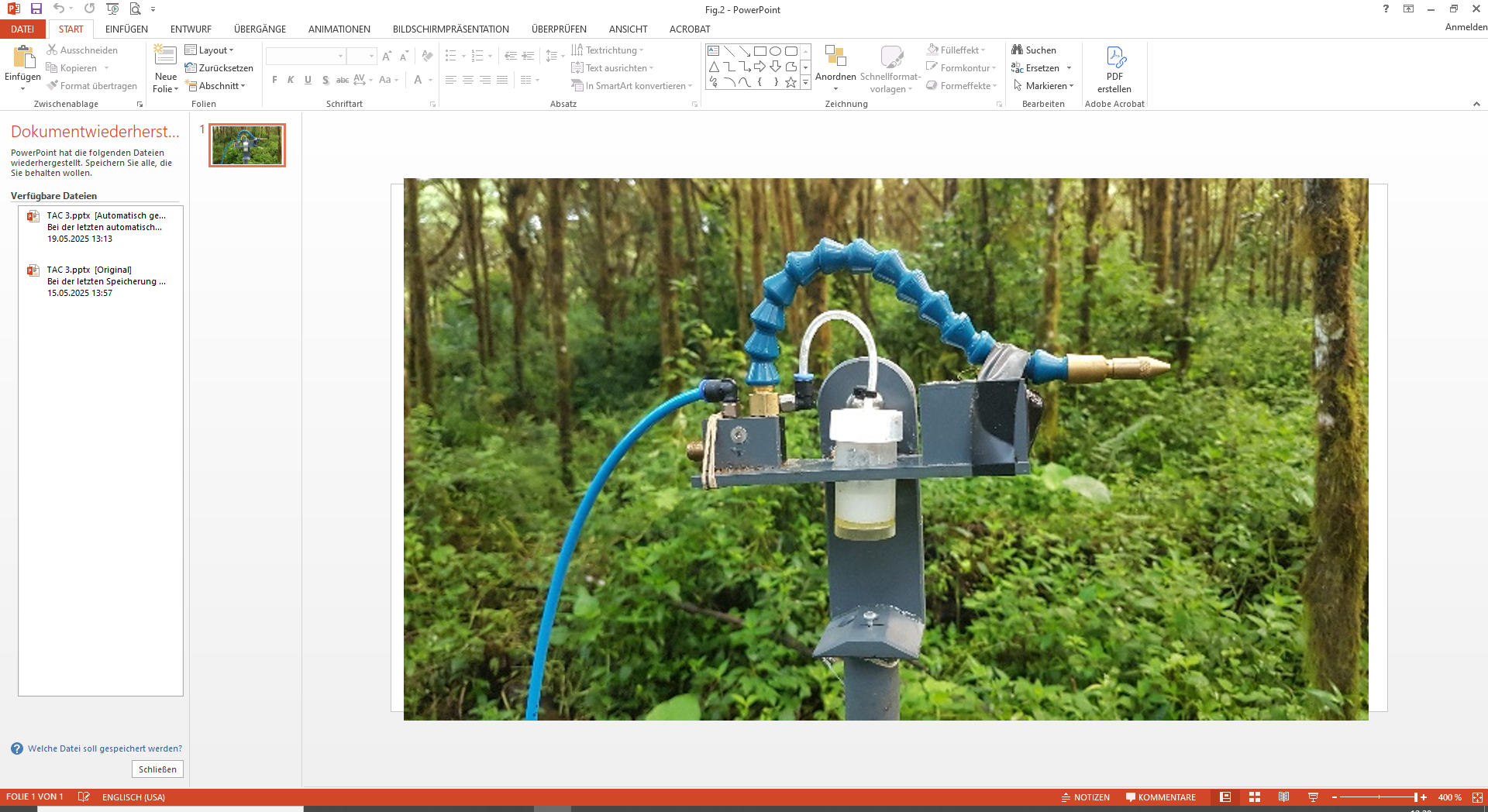


Fig. S1: Custom-built device used to apply insecticide to the nests using the Spritz technique. A compressor connected to the blue tube provided the airflow to spray the treatment liquid drawn up through the white tube. A wireless camera under the nozzle was used to monitor the application and ensure that the correct area was treated. Photo: MMF.


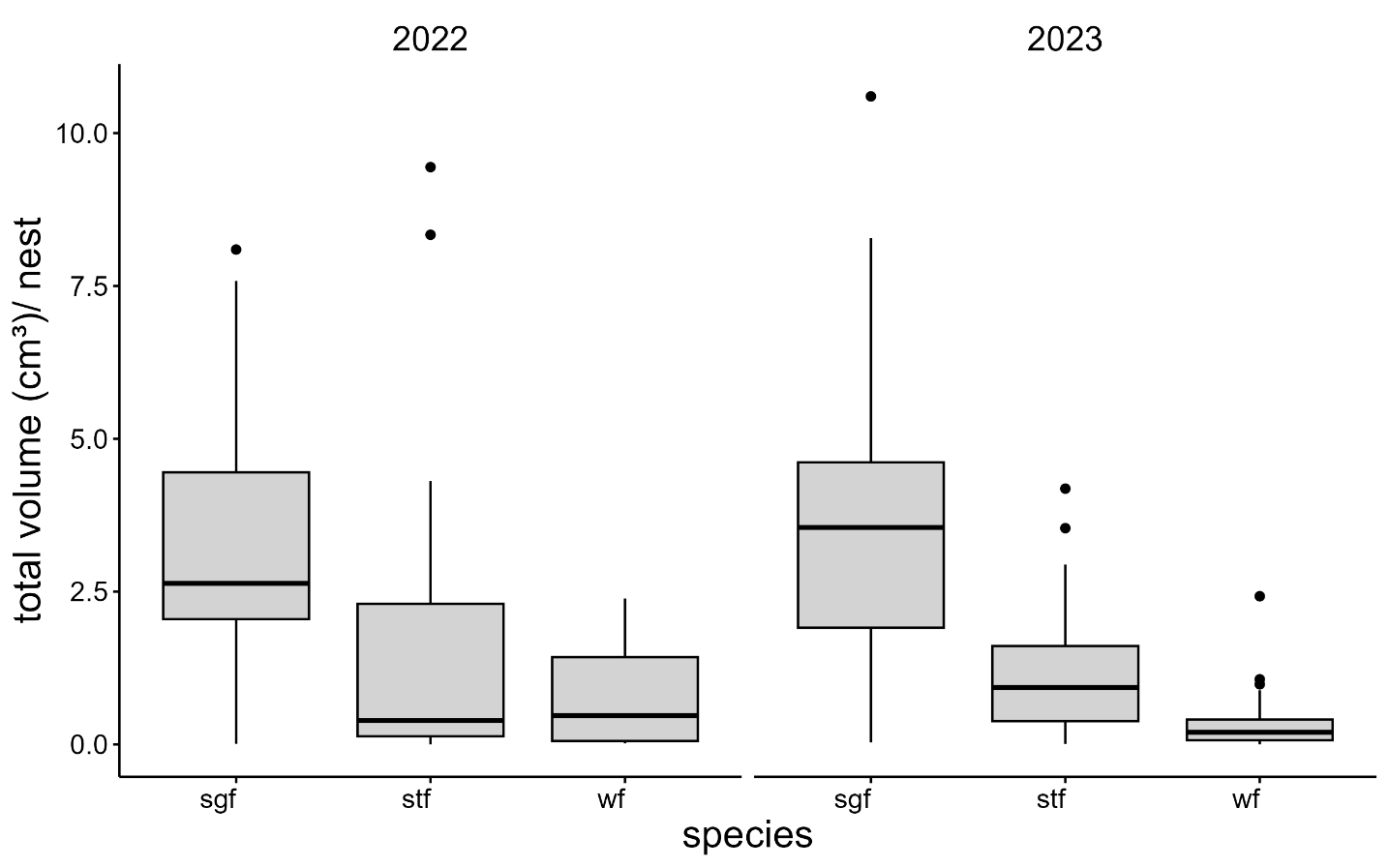


Fig. S2: Total volume of dispenser material (cm³) incorporated in nests of Small Ground-finches (sgf), Small Tree-finches (stf) and Green Warbler-finches (wf) in 2022 and 2023.


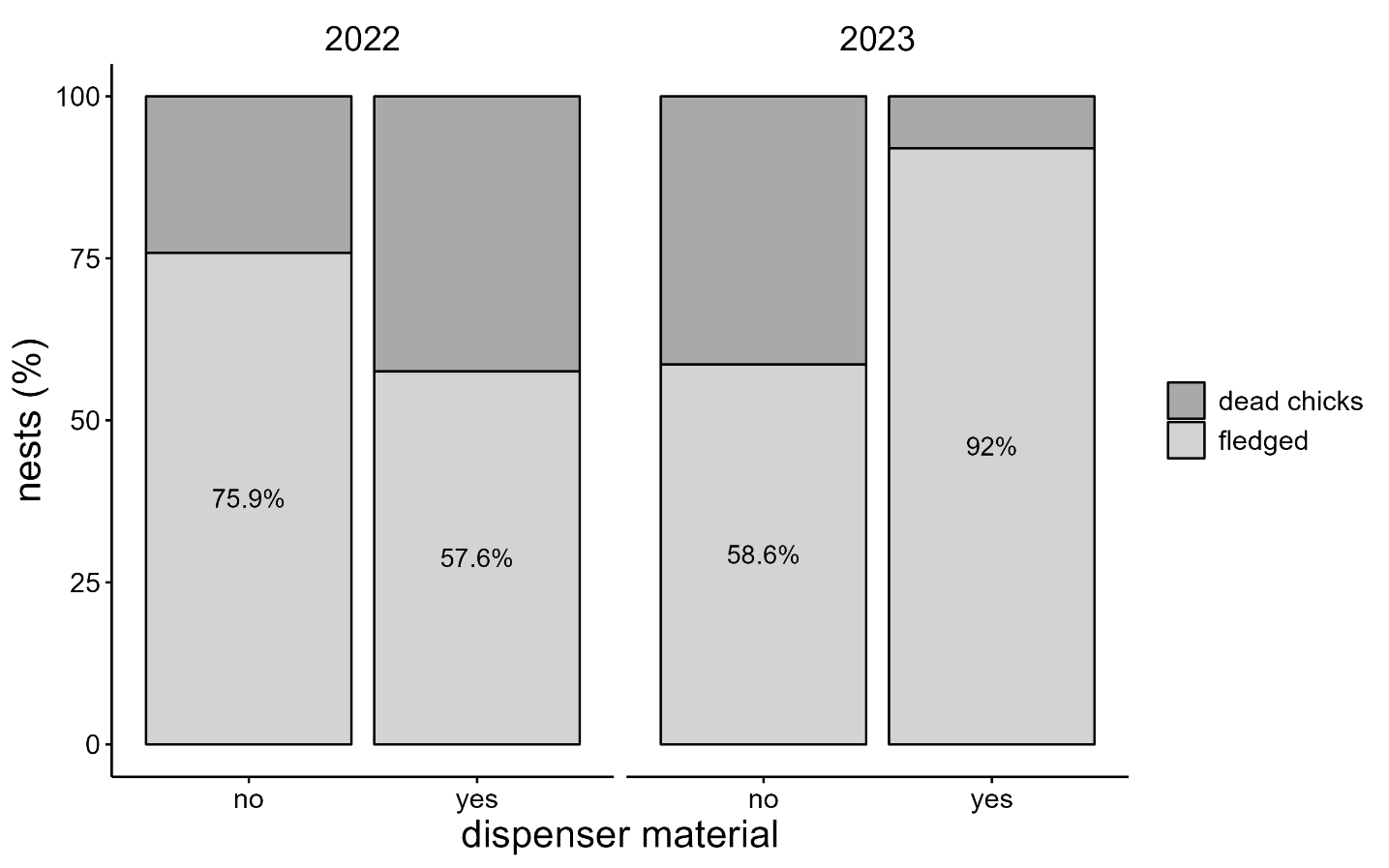


Fig. S3: Percentage of successful fledging in nests with and without dispenser material in 2022 (self-fumigation with Cyromazine) and 2023 (self-fumigation with 1% and 0.5% Permacap).


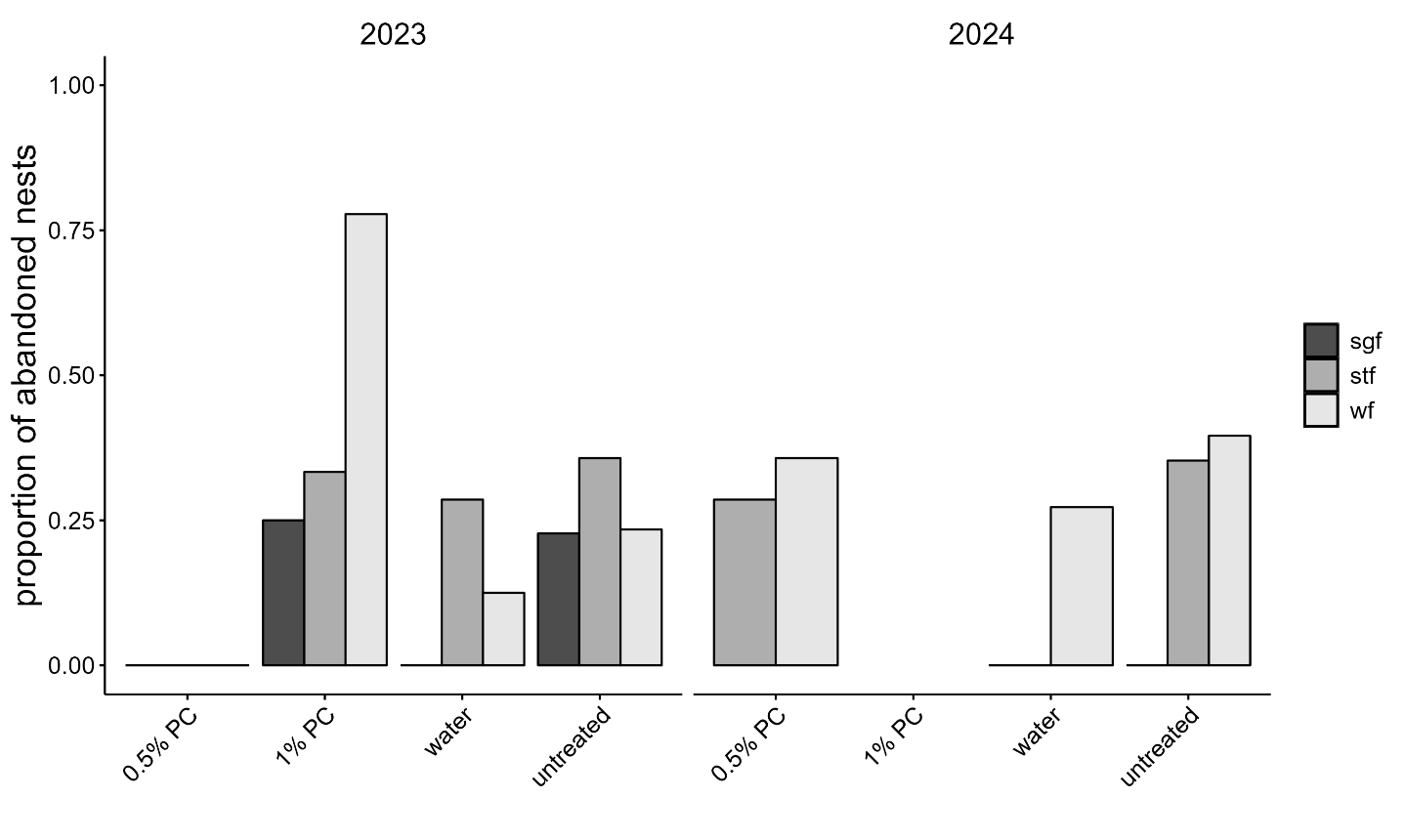


Fig. S4: Spraying treatment and nest abandonment rate (proportion) in Small Ground-finches (sgf), Small Tree-finches (stf) and Green Warbler-finches (wf) in the 2023 and 2024 experiment.
