# Supplementary material for: Innovations in invasive parasite control: enhancing nest treatment techniques to combat the threat of the avian vampire fly Philornis downsi in Galapagos
Source: Front Conserv Sci. Author manuscript; Available in PMC 2026 Jan 6. (PMC7618593; doi:10.3389/fcosc.2025.1591266)
Supplement: Supplementary Tables [file EMS211895-supplement-Supplementary_Tables.pdf]

Appendix

TABLE A1 Pairwise comparison of estimated marginal means (emmeans) for dispenser material prevalence across year-species combinations in Small Ground-finch (sgf), Small Tree-finch (stf) and Green Warbler-finch (wf) in 2022 and 2023; df = Inf. Asterisks indicate significance (* $p < 0.05$).

Contrast	Odds ratio	SE	Z-ratio	P-value
year2022 sgf/year2023 sgf	0.471	0.296	-1.199	0.838
year2022 sgf/year2022 stf	0.461	0.369	-0.969	0.928
year2022 sgf/year2023 stf	0.257	0.191	-1.826	0.449
year2022 sgf/year2022 wf	2.210	1.291	1.358	0.752
year2022 sgf/year2023 wf	1.321	0.743	0.495	0.996
year2023 sgf/year2022 stf	0.979	0.752	-0.028	1.000
year2023 sgf/year2023 stf	0.546	0.379	-0.872	0.953
year2023 sgf/year2022 wf	4.694	2.555	2.840	0.051
year2023 sgf/year2023 wf	2.805	1.396	2.072	0.302
year2022 stf/year2023 stf	0.558	0.482	-0.675	0.985
year2022 stf/year2022 wf	4.797	3.539	2.125	0.274
year2022 stf/year2023 wf	2.866	2.047	1.475	0.681
year2023 stf/year2022 wf	8.589	5.774	3.199	0.017 *
year2023 stf/year2023 wf	5.132	3.212	2.613	0.094
year2022 wf/year2023 wf	0.598	0.278	-1.107	0.879

TABLE A2 Pairwise comparison of estimated marginal means (emmeans) for dispenser material volume per nest across year-species combinations in Small Ground-finch (sgf), Small Tree-finch (stf) and Green Warbler-finch (wf) in 2022 and 2023. Asterisks indicate significance (* $p < 0.05$, *** $p < 0.001$).

Contrast	Estimate	SE	df	T-ratio	P-value
year2022 sgf - year2023 sgf	-0.3087	0.165	137	-1.873	0.4235
year2022 sgf - year2022 stf	0.6044	0.182	137	3.315	0.015 *
year2022 sgf - year2023 stf	0.5251	0.175	137	2.994	0.038 *
year2022 sgf - year2022 wf	0.9331	0.170	137	5.487	<.0001 ***
year2022 sgf - year2023 wf	1.0195	0.164	137	6.232	<.0001 ***
year2023 sgf - year2022 stf	0.9131	0.175	137	5.220	<.0001 ***
year2023 sgf - year2023 stf	0.8338	0.156	137	5.335	<.0001 ***
year2023 sgf - year2022 wf	1.2418	0.163	137	7.605	<.0001 ***
year2023 sgf - year2023 wf	1.3282	0.144	137	9.200	<.0001 ***
year2022 stf - year2023 stf	-0.0793	0.185	137	-0.429	0.998
year2022 stf - year2022 wf	0.3287	0.182	137	1.805	0.466
year2022 stf - year2023 wf	0.4151	0.174	137	2.389	0.167
year2023 stf - year2022 wf	0.4080	0.174	137	2.347	0.183
year2023 stf - year2023 wf	0.4944	0.155	137	3.186	0.022 *
year2022 wf - year2023 wf	0.0864	0.162	137	0.533	0.995

TABLE A3 GLM results, effect of volume (cm^3) of dispenser material with Cyromazine treatment on fledging success; reference category is Small Ground-finch (sgf) for species; (stf = Small Tree-finch, wf = Green Warbler-finch); N = 91; asterisks indicate significance (* $p < 0.05$)

Predictors	Estimate (95% CI)	SE	Z-value	P-value
(Intercept)	1.398 (0.211, 2.585)	0.606	2.309	0.021 *
z.volume	−0.047 (−0.659, 0.565)	0.312	−0.150	0.880
z.*P. downsi* abundance	0.221 (−0.294, 0.736)	0.263	0.840	0.401
species [stf]	−1.076 (−2.546, 0.394)	0.750	−1.435	0.151
species [wf]	−0.434 (−1.965, 1.096)	0.781	−0.556	0.578

TABLE A4 GLM results, effect of dispenser material prevalence (1% Permacap treatment) on fledging success; reference category is Small Tree-finch (stf) for species (sgf = Small Ground-finch, wf = Green Warbler-finch); N = 98; asterisks indicate significance (* $p < 0.05$, ** $p < 0.01$, *** $p < 0.001$)

Predictors	Estimate (95% CI)	SE	Z-value	P-value
(Intercept)	−1.972 (−3.684, −0.260)	0.873	−2.258	0.024 *
material [TRUE]	4.129 (2.020, 6.238)	1.076	3.837	<0.001 ***
z.*P. downsi* abundance	0.610 (−0.063, 1.282)	0.343	1.778	0.075
species [sgf]	2.101 (0.285, 3.917)	0.927	2.267	0.023 *
species [wf]	2.509 (0.685, 4.333)	0.931	2.697	0.007 **
